# Modification of the Histone Landscape with JAK Inhibition in Myeloproliferative Neoplasms

**DOI:** 10.3390/cancers12092669

**Published:** 2020-09-18

**Authors:** Graeme Greenfield, Suzanne McPherson, James Smith, Adam Mead, Claire Harrison, Ken Mills, Mary Frances McMullin

**Affiliations:** 1 Blood Cancer Research Group, Patrick G Johnston Centre for Cancer Research, Queen’s University, Belfast BT9 7AE, UK; g.greenfield@qub.ac.uk (G.G.); s.mcpherson01@qub.ac.uk (S.M.); jsmith41@qub.ac.uk (J.S.); k.mills@qub.ac.uk (K.M.); 2 Division of Genetics and Epidemiology, Institute of Cancer Research, London SW7 3RP, UK; 3 MRC Weatherall Institute of Molecular Medicine, University of Oxford, Oxford OX3 9DS, UK; adam.mead@imm.ox.ac.uk; 4 Department of Haematology, Guys and St Thomas Hospital, London SE1 9RT, UK; claire.harrison@gstt.nhs.uk; 5 Centre for Medical Education, Queen’s University Belfast, Belfast BT9 7BL, UK

**Keywords:** myeloproliferative neoplasms, JAK inhibition, epigenetics, histone modification

## Abstract

**Simple Summary:**

The introduction of JAK inhibitors has provided objective benefits to many patients with myeloproliferative neoplasms. However, the overall efficacy of these drugs is limited and improved therapeutic options are required. Epigenetic dysregulation is increasingly observed to play a role in these disorders. This study evaluates the changing landscape of histone modifications in myeloproliferative neoplasm (MPN) cell line models and patient samples from the MAJIC clinical trial following administration of the JAK inhibitor ruxolitinib, demonstrating that ruxolitinib has an epigenetic effect. This changing histone modification landscape may offer a target for further therapy to augment treatment.

**Abstract:**

Dysregulation of epigenetic processes is increasingly understood to play a role in the pathogenesis of myeloproliferative neoplasms (MPNs). Ruxolitinib, a JAK/STAT inhibitor, has proved a useful addition to the therapeutic arsenal for these disorders, but has limited disease modifying activity. We determined the effect of JAK inhibition on the histone landscape of MPN cells in cell line models of MPNs and validated using samples from the MAJIC randomised clinical trial of ruxolitinib in polycythaemia vera and essential thrombocythaemia. We demonstrated an epigenetic modifying effect of ruxolitinib using a histone modification assay. The majority of 21 histone H3 modifications were upregulated, with H3K27me3 and H3K36me2 significant in the combined cell line results. Chromatin immunoprecipitation and sequencing (CHIP-seq) for three marks of interest, H3K4me1, H3K4me3 and H3K27ac, was consistent with the histone modification assay showing a significant increase in H3K4me3 and H3K27ac peaks at promoter regions, both marks of active transcription. In contrast, RNA sequencing demonstrates a coordinated reduction in gene expression in a number of cell pathways including PI3K-AKT signalling, transcriptional misregulation in cancer and JAK-STAT signalling in spite of these histone changes. This highlights the complex mechanisms of transcriptional control within the cells which was reflected in analysis of the histone landscape in patient samples following ruxolitinib treatment.

## 1. Introduction

To understand the pathogenesis of the *BCR-ABL* negative myeloproliferative neoplasms (MPNs) solely on the basis of constitutive JAK/STAT signalling resulting from activating mutations in *JAK2* [1], *MPL* [2] or *CALR* [3] is an oversimplification. The complexity of the pathogenesis in these disorders is increasingly recognised to include dysregulation of normal epigenetic processes within the cell, the recurrent mutation of additional genes other than the driver genes and the environmental cellular interactions [4,5,6]. This complexity helps to explain and understand the heterogeneity of disease phenotype, progression risk and prognosis we recognise across this patient population in the MPN clinic [7,8].

Therapeutic options remain very limited. The introduction of ruxolitinib, a JAK1/2 inhibitor, has provided an additional line of therapy, effective at controlling haematocrit, reducing spleen volume and reducing the symptom burden in polycythaemia vera (PV) and primary myelofibrosis (PMF) [9,10,11]. However, in contrast to tyrosine kinase inhibitor therapy in chronic myeloid leukaemia, the disease modifying effects of ruxolitinib in MPN are modest. The *JAK2* V617F allele burden is variably and modestly reduced on therapy with sustained reductions evident on longer term therapy [12,13]. Stabilisation of bone marrow fibrosis can occur with therapy [14]. Rebound of disease on cessation of therapy is well recognised [15] and transformation to myelofibrosis or acute leukaemia in a high risk essential thrombocythaemia (ET) cohort was not ameliorated by ruxolitinib [16]. The benefit to overall survival has been debated [17]. It is clear that improving the therapeutic options for this heterogeneous patient group remains a major unmet need.

Control of transcription is a complex cellular process ultimately determining the differentiation and proliferation of the cell. Methylation of DNA and modification of histone lysine residues by methylation and acetylation are two of the more understood epigenetic processes impacting on individual gene transcription [18,19]. In MPN, DNA methylation patterns are abnormal in chronic phase disease and modified further in the transformation to blast phase disease [20]. A signature of methylation age is different between MPN disease phenotypes and can be modified by therapy [21]. Many genes involved in DNA methylation or regulation of histone modification are recurrently mutated in MPN and myeloid malignancy more generally including *TET2*, *DNMT3A*, *ASXL1* and *EZH2* [22,23]. Dysregulation of histone modification in MPN has been demonstrated [24]. Epigenetic modifying therapies including hypomethylating agents and histone deacetylase inhibitors have been trialled in MPN with some therapeutic efficacy noted [25,26].

Understanding the effect of JAK inhibition on the epigenetic landscape of the MPN cells may help to open an opportunity for augmenting treatment with targeted epigenetic therapies. The randomised control trial “MAJIC: A RandoMised study of best Available therapy versus JAK Inhibition in patients with high risk Polycythaemia Vera or Essential Thrombocythaemia who are resistant or intolerant to HydroxyCarbamide” (ISRCTN61925716) was designed to evaluate the efficacy of ruxolitinib in the second line setting in PV and essential thrombocythaemia (ET) against the best available therapy [27]. With the availability of pre- and post-treatment samples from this trial, we set out to establish the effects of ruxolitinib in comparison to the best available therapy for histone H3 modifications. Six histone modifications of interest established in cell line work were investigated with quantification of changes correlated with patient clinical and laboratory data.

## 2. Results

### 2.1. Effective Ruxolitinib Concentration in Cell Line Culture

We set out to investigate the epigenetic effect of ruxolitinib in MPN cell lines. An IC_50_ was established by examining the effect of ruxolitinib on cell viability. No clear cytotoxicity was seen, with the IC_50_ only being achieved after 72 h of treatment. This was established as 73 nM in the UKE-1 cell line, 55 nM in the SET-2 cell line and 325 nM in the HEL cell line. All doses were in the submicro molar range and well below the maximum serum concentration (Cmax) observed in a patient (maximum tolerated patient dose of 20 mg twice daily in a healthy subject resulted in a Cmax of 1160 nM). Western blotting showed inhibition of the phosphorylation of STAT3 and STAT5 at the 100 nM ruxolitinib concentration for UKE-1 and SET-1 cell lines. For the HEL cell line, a slightly higher concentration of ruxolitinib was required before this was clearly seen, at 250 nM. Ruxolitinib at a concentration of 100 nM was low enough not to induce cell death, but was sufficient to exert molecular responses in MPN cell lines with inhibition of the JAK/STAT pathway. All results available in Figure S1.

### 2.2. Histone Modification in Cell Culture

One of the commonest studied histone marks is H3K9. To establish if ruxolitinib treatment of MPN cell lines resulted in modification of histone H3, the UKE-1 cell line was treated with 100 nM of ruxolitinib over a time course. Western blots were performed utilising antibodies directed at H3K9 me1 (monomethylation at lysine 9), H3K9me3 (trimethylation at lysine 9) and H3K9ac (acetylation at lysine 9). An increase in methylation marks in ruxolitinib treated cells, maximal at 8 h, was observed. Acetylation levels were slightly reduced by 24 h. Total H3 and actin (used as a loading control) remained consistent throughout. The maximal increase in methylation was evident at 100 nM ruxolitinib when a range of dosing concentrations was used. This was consistent in the SET-2 and HEL cell lines. Collectively these results demonstrated that ruxolitinib treatment of MPN cell lines at this dosing concentration could induce the modification of histones. This is shown in Figure S2.

**Figure 1:**
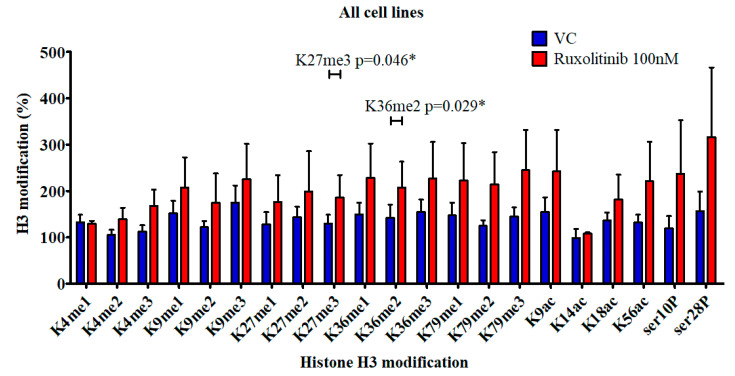
H3 modification as a percentage of total H3 present in myeloproliferative neoplasm (MPN) cell lines. * *p* value < 0.05.

A screen of 21 different histone H3 modifications allowed the effect of ruxolitinib treatment to be quantified. MPN cell lines were incubated with 100 nM ruxolitinib or equivalent volume of DMSO vehicle control (VC) for 8 h. In each of the cell lines, the majority of the marks were increased by ruxolitinib therapy with H3K27me3 (*p* = 0.04) significantly elevated in the UKE-1 cells. In the SET-2 cell lines, H3K4me1 and H3K4me3 were significantly increased (*p* = 0.026 and 0.031, respectively) whilst in the HEL cell line the elevation in H3K36me2 reached significance (*p* = 0.016). Each individual cell line is shown in Figure S3 whilst Figure 1 shows the results when all cell lines were combined. In this combined study, H3K27me3 and H3K36me2 show statistically significant elevations.

For the most significant marks to be selected for further analysis, the p values for each individual cell line, all cell lines combined and the cell lines in pairwise fashion were ranked in order of significance. The total sum of these rank orders was then generated. This data are displayed in Table S1. The top three most differentially modified by ruxolitinib therapy were H3K36me2, H3K36me1 and H3K4me2, in order. Methylation marks at lysine 36 ranked number 2, 1 and 10 (mono-, di- and trimethylation, respectively). Methylation marks at lysine 4 ranked 12, 3 and 5 (mono-, di- and trimethylation, respectively). Phosphorylation at serine 28 ranked number nine overall.

**Figure 2:**
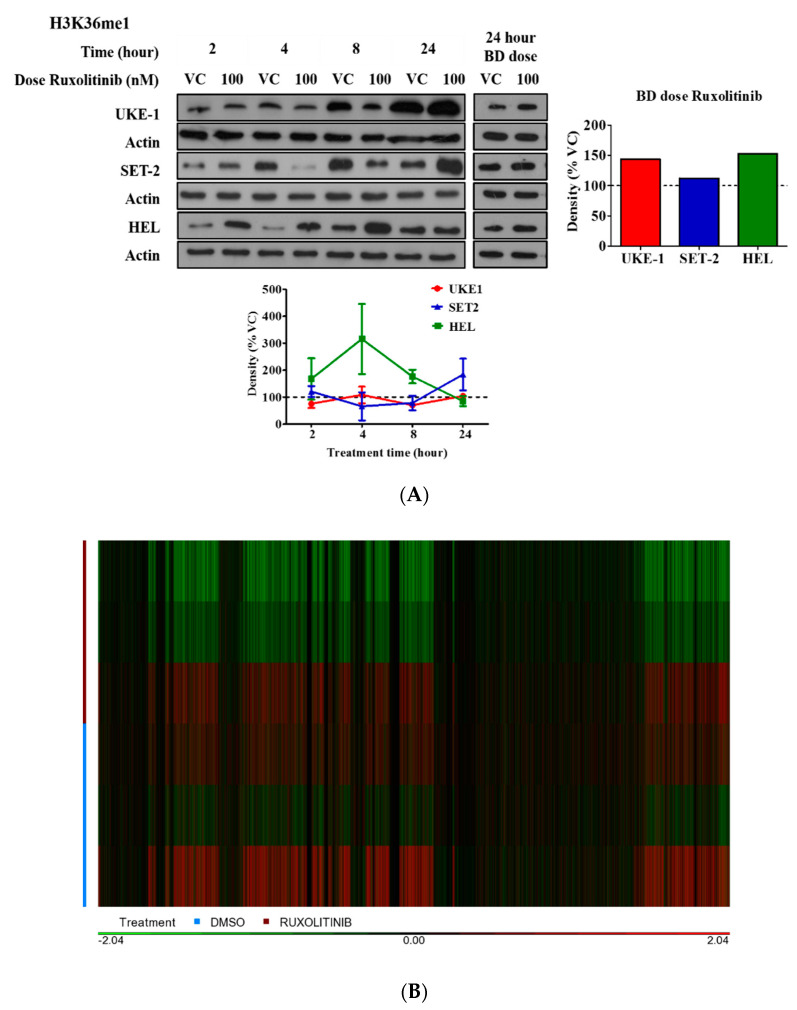

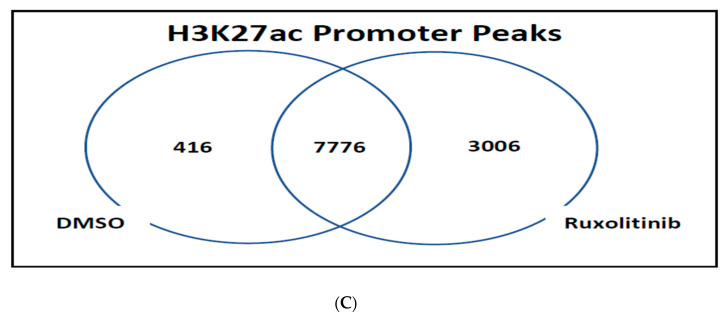
(**A**) Western blots for monomethylation levels at lysine 36 on histone H3 in MPN cell lines (UKE-1, SET-2 and HEL) treated with vehicle control or 100 nM ruxolitinib for 2, 4, 8 or 24 h (once or twice daily). Densitometry values are normalised for loading control. (**B**) A heat plot comparing global gene expression from RNA seq analysis for ruxolitinib and DMSO treated HEL cells. (**C**) A Venn diagram demonstrating the number of exclusive H3K27ac peaks at gene promotor regions in cells treated with ruxolitinib or DMSO control. The middle represents the common peaks between the two treatments.

Subsequently these seven modifications were taken forward for validation. MPN cell lines were treated with 100 nM ruxolitinib or an equivalent volume of VC. In addition, the effect of mimicking a patient dosing regime was examined with cells dosed twice with 100 nM of ruxolitinib or VC in a 24 h period. The entire cell suspension was pelleted at 24 h and Western blots performed for each mark. The modification of each mark with ruxolitinib therapy varied between cell lines and over time. However, by mimicking a patient dosing regimen, the marks were more consistently increased. Figure 2A displays the results for H3K36me1. The remaining results are available in Figure S4.

The pattern of histone modifications by time point, normalised for loading control for H3K4 and H3K36, is shown in Figure S5A. Finally, the results of the histone arrays and Western blot validation were correlated. An analysis of variance (ANOVA) was undertaken using the Partek Genomics Suite 6.0 Software. The two techniques were positively correlated with an R^2^ value of 0.7394 generated. This is demonstrated in Figure S5B.

To understand the significance of these histone modifications in greater detail, chromatin immunoprecipitation and sequencing (CHIP-seq) was performed for three histone marks of primed and active transcription (H3K4me1, H3K4me3 and H3K27ac) alongside RNA sequencing. HEL cells were treated with 100 nM ruxolitinib or an equivalent volume of vehicle control twice at 12 h intervals and harvested at 24 h post the initial dose. Analysis of differential gene expression from RNA-seq demonstrated a coordinated reduction in expression (Log2FC < −0.5, *p* value < 0.05) of 120 genes in the treated cells in comparison to the control. The heatmap is shown in Figure 2B. Pathway analysis using STRING shows enrichment of genes involved in PI3K-AKT signalling, transcriptional misregulation in cancer and JAK-STAT signalling pathway genes. The full list of affected pathways is available in Table S2 [28].

CHIP-seq peaks at promoter regions were determined for the three histone marks as described. A clear differential was evident for H3K4me3 and H3K27ac, two marks of active transcription. In keeping with the histone array assay, a marked increase in H3K4me3 peaks was seen across the genome whilst the difference between H3K4me1 was much more subtle. For H3K4me3, 3702 peaks at promoter regions were only seen in the treated cells whilst only 33 peaks were present in control cells. When H3K27ac was examined, 3006 promoter peaks were seen only in the treated cells whilst only 416 promotor peaks were exclusive to the control cells. A total of 7776 H3K27ac promotor region peaks were common between ruxolitinib treatment and DMSO control. This is shown in Figure 2C. For H3K4me1, a mark of primed transcription, 33 peaks were only present in treated cells in comparison to seven peaks in control cells only. 

### 2.3. Examination of Candidate Histone Modifications in Patient Samples from the MAJIC Clinical Trial

We then set out to examine the effective of ruxolitinib therapy on targeted histone modifications in MPN patients on a clinical trial and investigate if any associations with clinical outcomes were evident. “MAJIC” (ISRCTN61925716) is a randomised study of best available therapy (BAT) versus *JAK* inhibition in patients with high risk PV or ET, who are resistant to or intolerant of hydroxycarbamide. BAT was assigned according to the physician’s preference but had to be an active agent. No crossover of BAT to ruxolitinib was permitted and no JAK inhibitors were used as BAT. An alteration of BAT therapy was permitted within the trial. Within each disease arm of the MAJIC trial (MAJIC-PV and MAJIC-ET), an independent, parallel, open-label, randomised controlled trial of ruxolitinib versus BAT was carried out. Patients were stratified by *JAK2* status and then randomised on a 1:1 ratio to receive either ruxolitinib or BAT. The candidate histone modifications found to be most significantly altered by ruxolitinib therapy in cell lines detailed above were taken forward into patient samples from the MAJIC clinical trial. Additional ethical approval was received for this work.

Peripheral blood (PB) or bone marrow (BM) granulocyte pellets were available at trial entry and after a period on therapy (both treatment arms and disease groups). To quantify the change in histone marks occurring during trial follow up, Western blotting with antibodies directed at six histone modifications of interest (mono-, di- and trimethylation at lysine 4 and 36 on histone H3) on extracted protein from trial entry and follow up samples. These data were then correlated with clinical information such as age, gender, disease group, treatment arm, response criteria and thrombosis and adverse event rates.

**Figure 3:**
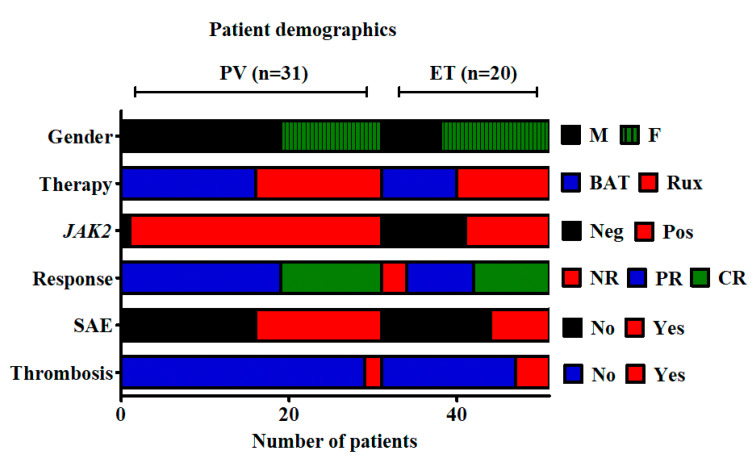
MAJIC patient demographics. Gender, treatment arm, *JAK2* status, response status and incidence of serious adverse events (SAEs) and thrombosis per disease arm for the MAJIC cohort examined.

Paired samples on 51 patients from the MAJIC trial were analysed. These were timed at trial entry (TE) and at a median follow-up (FU) time of 13 months (range 9–36). These samples (*n* = 101) were in the form of granulocyte pellets; 91 originating from peripheral blood (PB) and 11 from bone marrow (BM). Patient demographics are demonstrated in Figure 3. Of the 10 *JAK2V617F* negative patients, five had detectable mutations in *CALR* (50%) and one in *MPL* (10%). Unfortunately, the presence of any additional high-risk mutations was unavailable in either disease group at time of analysis.

**Figure 4:**
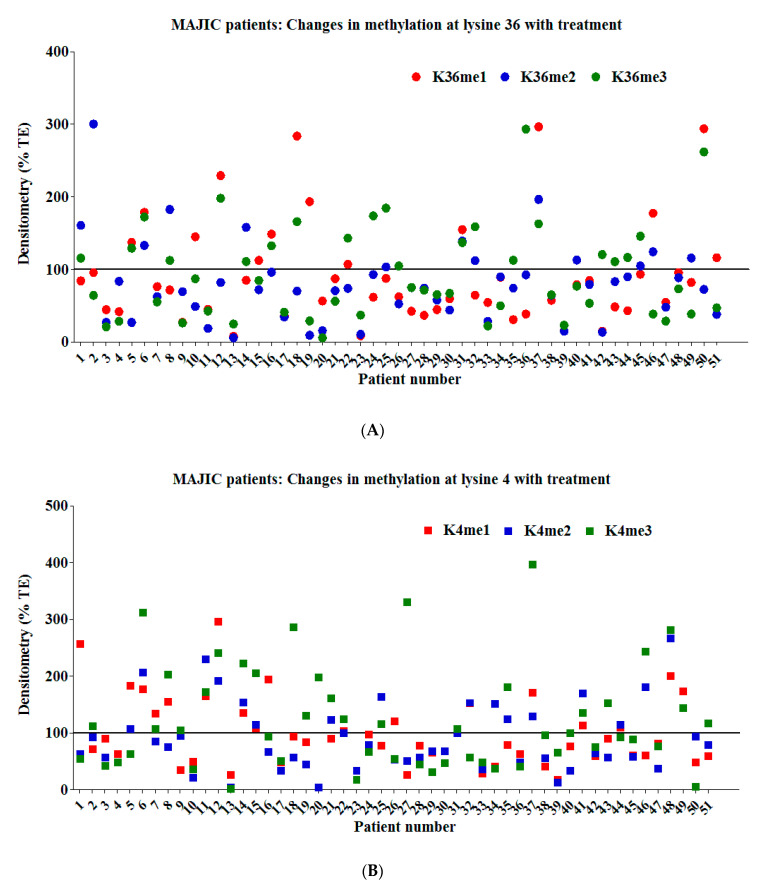
(**A**) MAJIC patients, modification of marks at lysine 36 in follow-up samples. For all 51 patients examined, the percentage change in each form of methylation at lysine 36 on histone H3. (**B**) MAJIC patients, modification of marks at lysine 4 in follow-up samples. For all 51 patients examined, the percentage change in each form of methylation at lysine 4 on histone H3.

Figure 4A,B show the percentage change in normalised densitometry values for each form of methylation for all 51 patients at lysine 36 and lysine 4, respectively. For some, all forms of methylation increased with treatment, for others all forms decreased, for still others one or two forms increased, while the other form of methylation decreased. Individual Western blot data are available in Figure S6. There was no apparent pattern between patients. The mutational allele burden offers a surrogate for the size of the neoplastic clone at diagnosis and follow-up. However, at time of analysis, the mutational allele burden was available in only 16/51 patients. Using a two-tailed paired t test, there was no significant allele burden reduction in the patients (*p* = 0.44) across the whole cohort, whilst the nine patients with available data in the ruxolitinib arm just reached statistical significance (*p* = 0.04) due to a significant reduction in the *JAK2* V617F burden in one patient in this arm. Removing this one patient from the analysis of this arm resulted in a loss of the statistical significance (*p* = 0.14). This suggests that the neoplastic clone size is likely to be similar at TE and FU and does not account for the changing methylation landscape.

### 2.4. Correlation of Histone Modifications with Treatment Response

**Figure 5:**
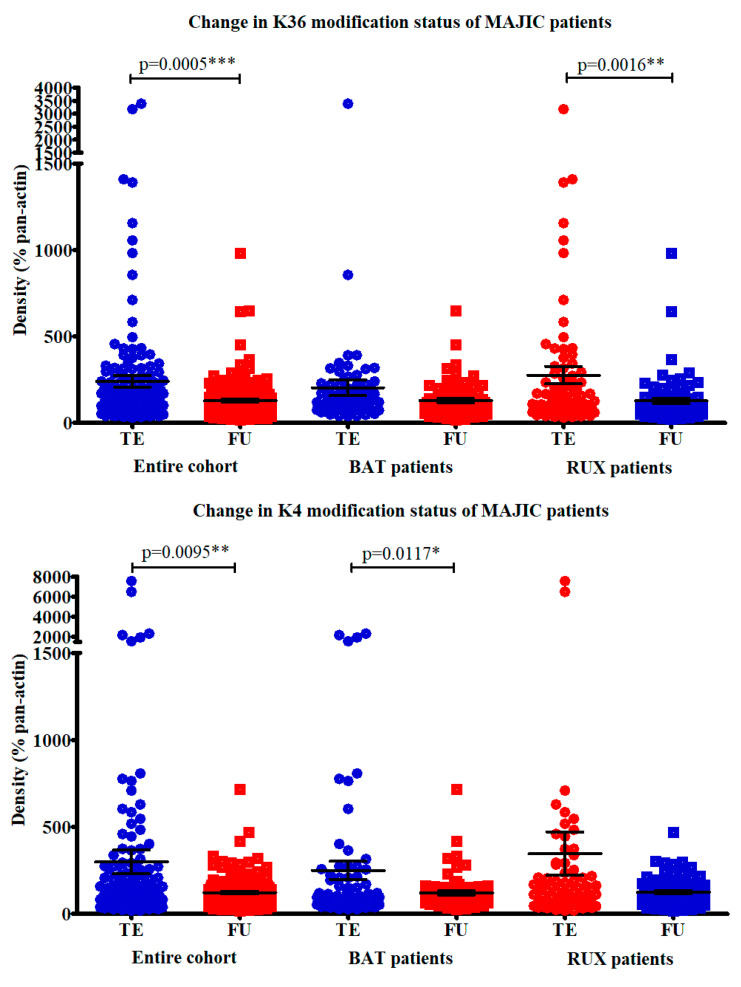
Change in histone modification status of MAJIC patients—both sets of histone modifications were significantly decreased in follow-up (FU) samples, compared to those at trial entry (TE). * *p* value < 0.05, ** *p* value < 0.01, *** *p* value < 0.001.

The change in each modification was examined in turn, to determine any links with the disease group or treatment arm. There was no change in any histone mark that was associated with the treatment arm of the patients in either disease group. In addition, multivariate analysis did not reveal any correlations between the change in histone modifications and gender, age, *JAK2* status, rates of thrombosis or SAEs. When all the modifications within the same lysine where examined together (i.e., mono-, di-, and trimethylation), both sets of methylation marks were significantly decreased in FU samples, compared to TE, with densitometry values normalised for pan-actin used as a loading control (H3K36 *p* = 0.0005, H3K4 *p* = 0.0095). The decrease in H3K36 marks was also significant when the ruxolitinib patients were examined separately (*p* = 0.0016), but not in the BAT cohort. On the other hand, the decrease in H3K4 marks was significant in the BAT patients when the groups were separated (*p* = 0.0117), but not in the ruxolitinib patients. Figure 5 shows this. Figure S7 demonstrates the change observed in each individual patient.

**Figure 6:**
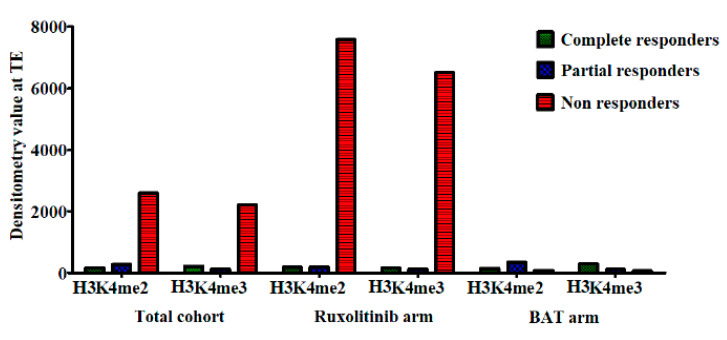
H3K4 levels at trial entry correlate with response.

A multivariate analysis was performed to see if the change in any of the candidate histone modifications was associated with clinical response. No such associations were found. Finally, the baseline and follow-up levels for each mark were examined in isolation. Although the cohort size was small (*n* = 3/51), high TE levels of H3K4me2 and H3K4me3 were significantly associated with a non-response to ruxolitinib (*p* < 0.0001). We could not determine an association with allele burden due to the low number of patients. This is demonstrated in Figure 6.

Non-responders (NR) had significantly higher baseline levels of di- and trimethylation at lysine 4 on histone H3.

## 3. Discussion

The JAK1/2 inhibitor ruxolitinib has proved to be a useful addition to a very limited therapeutic arsenal in the management of MPNs [11]. Understanding the effects of therapy on the complex cellular processes driving the transcriptional machinery of the MPN cell may offer an insight into potential targets for augmenting therapy. It is increasingly clear that dysregulation of normal epigenetic processes contributes to the pathophysiology of these disorders [4,29]. The significant heterogeneity in MPN mean that cell lines offer an imperfect solution for studying disease biology. It is impossible that a single cell line can fully reflect the disease biology of both a stable patient with chronic phase ET and a patient with advanced myelofibrosis or blast phase disease simultaneously. However, in this study, using three separate cell lines with a different mutational spectrum and heterozygosity or homozygosity for the *JAK2* V617F mutation we have been able to draw meaningful comparisons of the epigenetic response to JAK inhibitors. We have demonstrated that ruxolitinib has an immediate impact on the histone landscape in MPN cell line models and can modify this landscape in samples from patients on this therapy. These effects are most in keeping with a brake on the transcriptional drive of JAK/STAT signalling but may also reflect some off-target effects of the drug. In our cell line work, we have demonstrated that, within a short time of ruxolitinib administration, there is a significant increase in the methylation, acetylation and phosphorylation of many histone H3 modifications. Many of these marks, including the H3K4me3 mark examined with CHIP-seq, are marks of actively transcribed genes. Yet, the transcriptome of the cell shows a clear coordinated reduction in the expression of genes involved in critical cell signalling pathways with few upregulated genes in response to ruxolitinib. Therefore, the immediate epigenetic repriming of the cell by ruxolitinib is not immediately reflected in the transcriptome. This highlights the complex nature of transcriptional control within these cells. This upregulation of the histone marks of active transcription may reflect a feedback mechanism within the cell to return to the “new normal” of enhanced transcription driven by constitutive JAK/STAT activation. Understanding the dynamics and evolution of these histone modifications over longer time periods will be important.

In the patient samples, the picture, as in the clinic with a heterogeneous patient population, is equally complex. The MAJIC trial reported no benefit of ruxolitinib in the ET cohort which contributed 20 of the 51 samples available in this study. This reflection on the biological differences between PV and ET may mask any significant findings within our own study. We observed that methylation patterns of H3K4 and H3K36 were variable between patients and altered by therapy with a decrease in marks observed on ruxolitinib and BAT. The upregulation of histone modifications seen in the cell line work were not evident. This evolving histone landscape over months of therapy suggests a dynamic process of transcriptional control which appears to be more reflective of the role of therapy by modifying transcription to repress proliferation. Understanding how these global changes in the histone landscape are reflected on an individual gene basis with linked transcriptomic data, particularly in the disease initiating and sustaining stem and progenitor cell populations, may offer opportunity for targeted therapy.

Although limited by sample size, our data also suggested that high baseline levels of di- and trimethylation at lysine 4 appeared to be linked with non-response to ruxolitinib. This finding requires prospective validation in a larger cohort but may indicate that, in some patients, the effects of JAK inhibition cannot overcome the cellular processes driving proliferation reflected by this epigenetic landscape. This may be a potential guide to understand which patients are likely to benefit from JAK inhibition prior to commencing therapy. 

## 4. Materials and Methods

### 4.1. Cell Culture and Drug Treatment

Three cell lines, UKE-1, SET-2 and HEL, were used and cultured using a standard technique with RPMI 1640 media supplemented with 20% foetal bovine serum (UKE-1 and SET-2) or 10% foetal bovine serum (HEL) with 1% penicillin/streptomycin. Stock concentrations of ruxolitinib were prepared and diluted to desired concentrations. These were added to standardised volumes of cell suspension at 2 × 10^5^/mL. Ruxolitinib was solubilised in DMSO not exceeding 0.1%. DMSO, at 0.1%, was dosed as the vehicle control. Cell viability was assessed using Cell titre-glo luminescent cell viability assay as per the manufacturer’s instructions. IC_50_ was determined using GraphPad Prism version 5 software.

### 4.2. Western Blotting

Extraction buffer comprised of radioimmune precipitation assay buffer, protease inhibitor and phosphatase inhibitor was used on cell pellets with approximately 1 × 10^6^ cells. Protein concentration of the lysate was determined using Pierce BCA protein assay kit. In total, 25 ng of total protein per well was added to 10× loading buffer prior to denaturing at 95 °C for 5 min. Protein was stored at −20 °C prior to use. Gels were hand cast at a concentration of 12–15%. A total of 25 ng of protein was added to each well and electrophoresis was undertaken in a Bio-Rad electrophoresis tank. A current of 15 mAmp per gel was applied for 1 h 20 min. Protein was then transferred to a nitrocellulose membrane with pore size of 0.2 µm prior to Ponceau S staining, Tris buffered saline-Tween (TBST) wash and membrane block in 5% milk and incubation with primary antibody overnight at 4 °C. Further washing with TBST was conducted, then incubation by conjugated antibody for 1 h prior to addition with chemiluminescence substrate and development of photographic film in a dark room. ImageJ software was used to obtain densitometry readings on each blot of interest.

### 4.3. Histone Modification Assay

Histones were extracted using the Epiquik Histone extraction kit as per the manufacturer’s instructions. Extracts were stored at −20 °C until used. The levels of 21 histone H3 modifications were measured using the EpiQuik™ Histone H3 modification multiplex assay kit (colormetric) as per the manufacturer’s instructions. 

### 4.4. RNA and Chromatin Immunoprecipitation(CHIP) Sequencing

RNA was extracted from cell pellets using a Qiagen RNeasy kit as per the manufacturer’s instructions. RNA library preparation with riboerase and sequencing on an Illumina NEXTseq platform undertaken by Genomics Core Technology Unit, Queen’s University Belfast was conducted. RNA sequencing was carried out at 75 BP reads with 15 M reads per sample. Normalised gene counts were analysed using DESeq2 to establish differential gene expression. STRING database was used to undertake pathway analysis [28].

For CHIP-seq, cells were fixed with 1% formaldehyde at room temperature for 10 min before quenching with 0.25 M glycine. Cells were washed with PBS and lysed with cytoplasmic lysis buffer followed by nuclear lysis buffer. The resulting chromatin pellet was suspended in sonication buffer prior to sonication with the Bio-ruptor UCD 500 to an average size of 200–500 BP. Chromatin was precleared for 1 h prior to overnight incubation with Dynabeads Protein A/G coupled to 5 µg of the target antibody or IgG. The following day beads were washed with low salt, high salt and lithium chloride buffers before resuspension in tagmentation buffer. A total of 1 μL Tagment DNA Enzyme from the Nextera DNA Sample Prep Kit was added and incubated at 37 °C for 10 min in a thermocycler. Tagmented DNA was eluted from beads using elution buffer and amplified with Nextera Primers and KAPA HiFi for 5–10 cycles before sequencing was undertaken by the Genomics Core Technology Unit, Queen’s University Belfast on an Illumina NextSeq 500 platform. Resulting FASTQ files were concatenated, trimmed with Trim Galore! prior to genome alignment with BOWTIE2 on the usegalaxy.eu platform [30]. Differential peak analysis was undertaken using the CHIP-seq pipeline available on the Partek Genomics Suite Software. Figure S8 details the CHIP-seq analysis pathway.

### 4.5. Ethical Approval

A favourable ethical opinion was obtained from the North West–Greater Manchester West Research Ethics Committee (16/NW/0191) covering use of all patient material in this study.

## 5. Conclusions

Just as the pathogenesis of MPNs is more complex than the simple constitutive activation of JAK/STAT signalling, the efficacy of JAK inhibition appears to result from a wide range of effects including an evolving histone landscape. Understanding the complexities of the epigenetic effects of therapy may help to provide an insight into future effective therapeutic approaches with targeted epigenetic modifiers to augment JAK inhibition.

## Supplementary Materials

The following are available online at https://www.mdpi.com/2072-6694/12/9/2669/s1: Figure S1A: UKE-1, SET-2 and HEL cells incubated with doses of Ruxolitinib and cell viability assessed by CTG^®^ after 24, 48 and 72 h, Figure S1B: UKE-1, SET-2 and HEL cells treated for 24 h with doses of Ruxolitinib and protein extracted. Figure S2: Protein extracted from vehicle control or 100 nM Ruxolitinib treated UKE-1 cells at 2, 4, 8 and 24 h. Figure S3: Quantity of H3 modification as a percentage of total H3 present in UKE-1, SET-2 and HEL cell lines. Figure S4: Western blots for H3K36me2, H3K36me3, H3ser28P, H3K4me1, H3K4me2 and H3K4me3 Histone H3 modifications in MPN cell lines (UKE-1, SET-2 and HEL) treated with vehicle control or 100 nM Ruxolitinib for 2, 4, 8 or 24 h (once or twice daily). Figure S5A: Pattern of Histone H3 modifications in MPN cell lines with varying duration of Ruxolitinib treatment. Figure S5B: Densitometry values from Western blots correlated with histone array values for each histone H3 modification tested. Figure S6: Western blots for mono-, di- and trimethylation at lysine 36 and lysine 4 on histone H3 and corresponding pan-actin for all 51 MAJIC patients examined. Figure S7: For each patient the normalised densitometry values before and after therapy for each histone mark were examined. Figure S8: CHIP-seq analysis pipeline. Table S1: Histone arrays, rank order of p values, Table S2: STRING pathway analysis of downregulated genes following ruxolitinib administration to HEL cells (2).
