# Supplementary material for: Modification of the Histone Landscape with JAK Inhibition in Myeloproliferative Neoplasms
Source: Cancers (Basel). 2020 Sep 18;12(9):2669. doi: 10.3390/cancers12092669 (PMC7563593; doi:10.3390/cancers12092669)
Supplement: Supplementary file 1 [file cancers-12-02669-s001.pdf]

cancers

Article

Modification of the Histone Landscape with JAK Inhibition in Myeloproliferative Neoplasms

Graeme Greenfield [1,†], Suzanne McPherson [1,†], James Smith [1,2], Adam Mead [3], Claire Harrison [4], Ken Mills [1] and Mary Frances McMullin [5,*]

1 Blood Cancer Research Group, Patrick G Johnston Centre for Cancer Research, Queen's University, Belfast, BT9 7AE, United Kingdom; g.greenfield@qub.ac.uk (G.G.); s.mcpherson01@qub.ac.uk (S.M.); jsmith41@qub.ac.uk (J.S.); k.mills@qub.ac.uk (K.M.)
2 Division of Genetics and Epidemiology, Institute of Cancer Research, London, SW7 3RP, United Kingdom
3 MRC Weatherall Institute of Molecular Medicine, University of Oxford, Oxford, OX3 9DS, United Kingdom; adam.mead@imm.ox.ac.uk
4 Department of Haematology, Guys and St Thomas Hospital, London, SE1 9RT, United Kingdom; claire.harrison@gstt.nhs.uk
5 Centre for Medical Education, Queen's University Belfast, Belfast, BT9 7BL, United Kingdom; m.mcmullin@qub.ac.uk
* Correspondence: m.mcmullin@qub.ac.uk
† Joint First Author.

Received: 20 August 2020; Accepted: 15 September 2020; Published: date

Supplementary materials

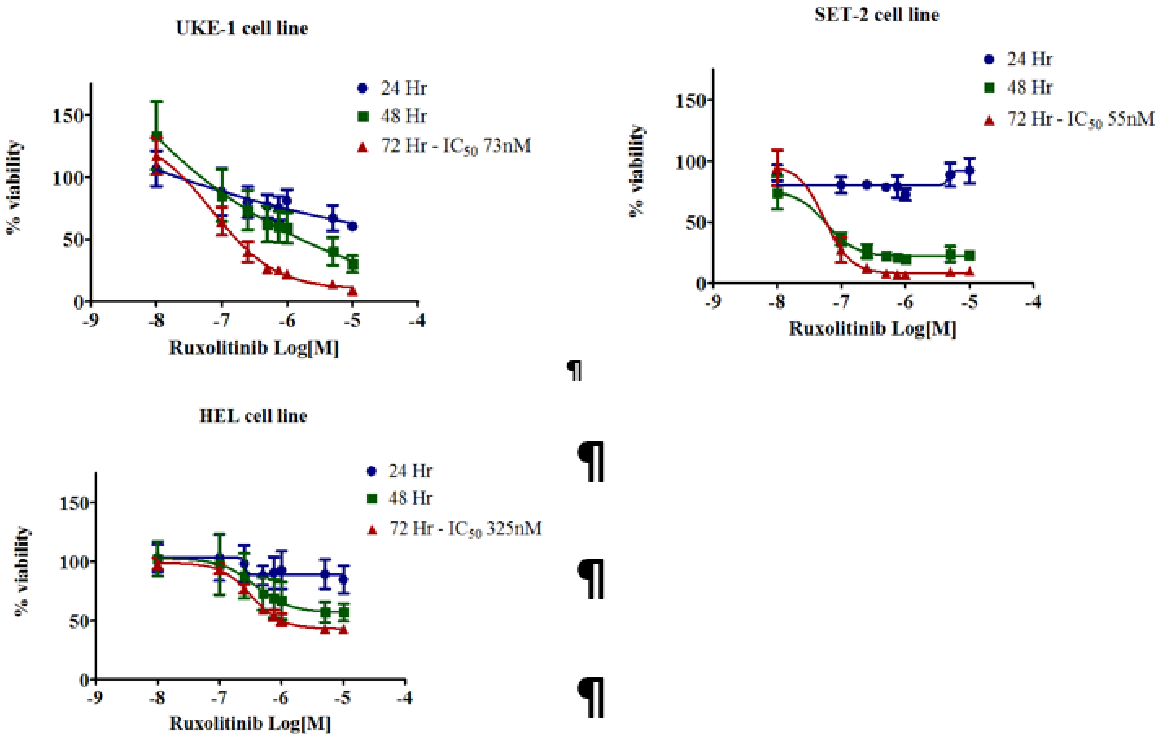

¶

¶

¶

¶

(A)

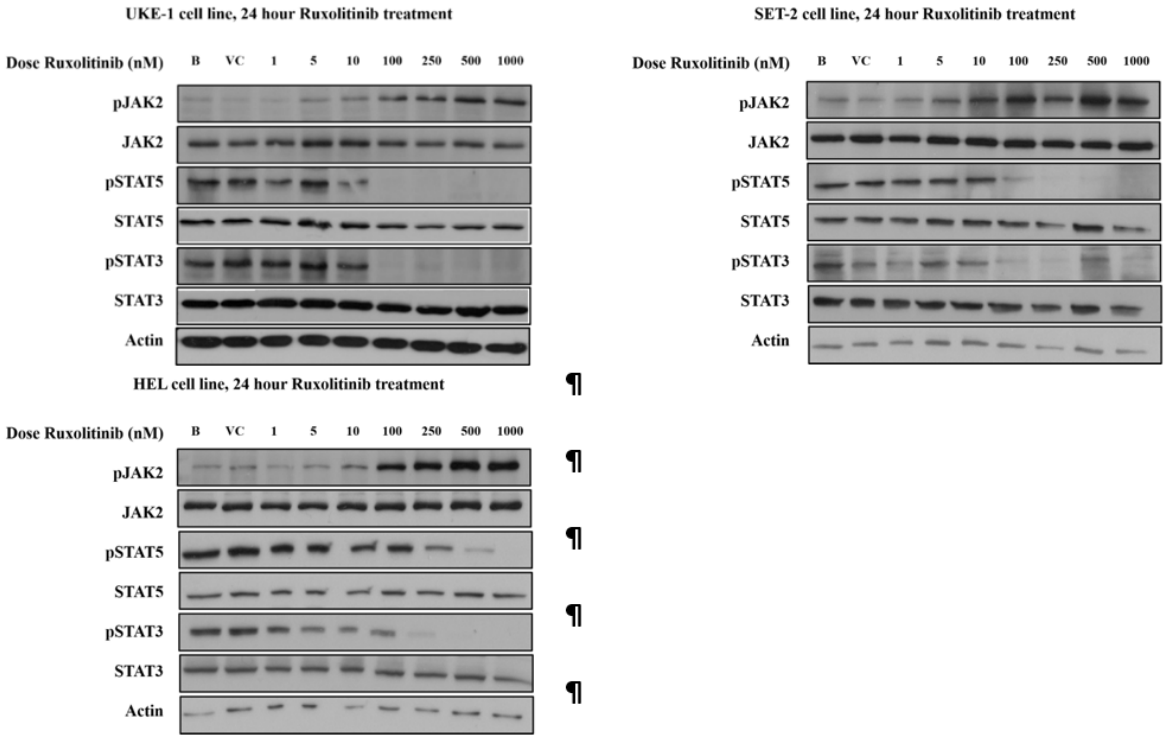

(B)

Figure 1. (A) UKE-1, SET-2 and HEL cells incubated with doses of Ruxolitinib and cell viability assessed by CTG® after 24, 48 and 72 hours. The IC_{50} was calculated using GraphPad Prism software. **(B)** UKE-1, SET-2 and HEL cells treated for 24 hours with doses of Ruxolitinib and protein extracted. Western blotting performed utilizing antibodies directed at the JAK/STAT pathway.

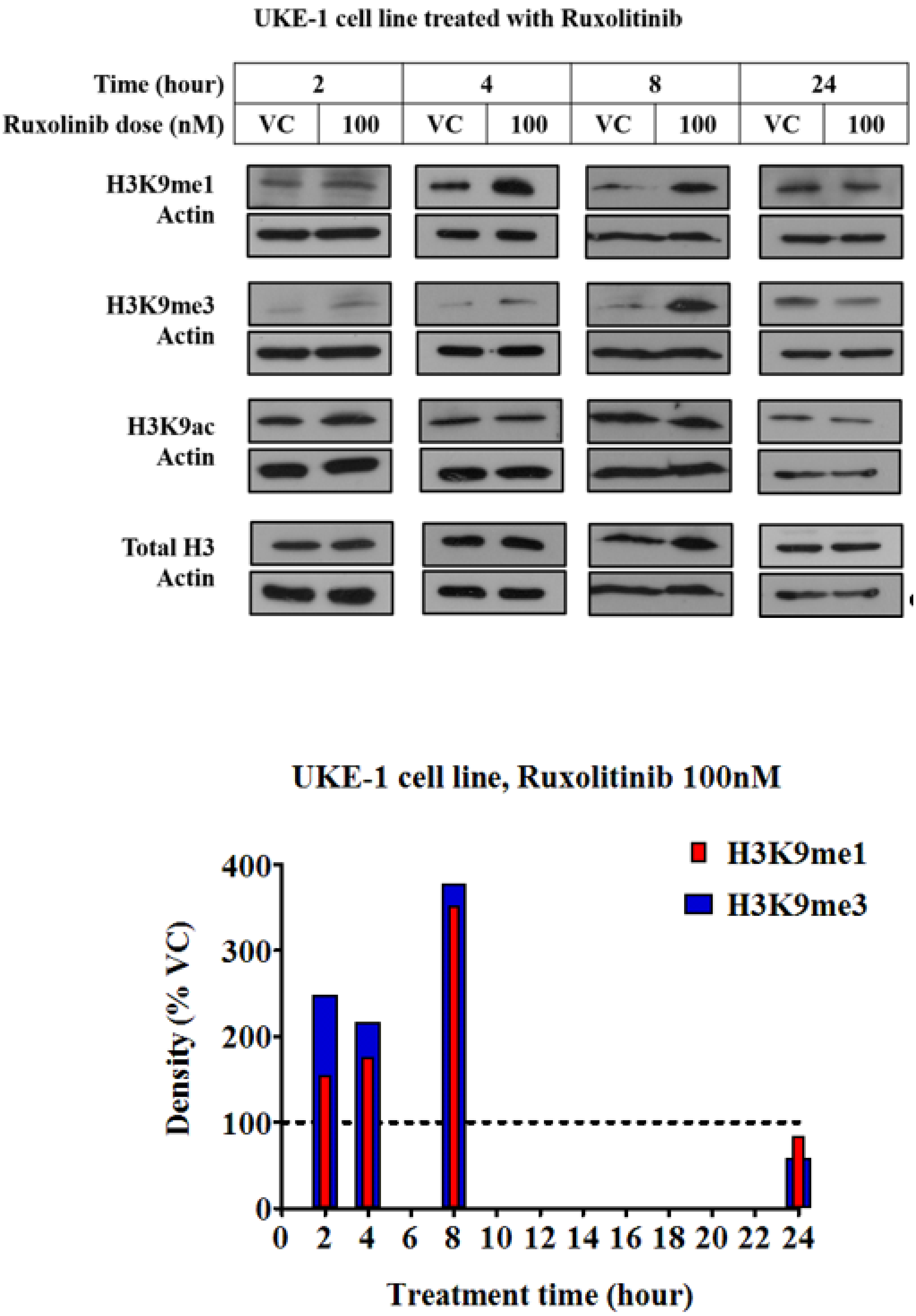

Figure 2. Protein extracted from vehicle control or 100nM Ruxolitinib treated UKE-1 cells at 2, 4, 8 and 24 hours. Western blots performed to look for effects on Histone H3 with densitometry confirming an increase in mono- and tri-methylation at lysine 9 in treated cells. Acetylation levels were slightly reduced by 24 hours.

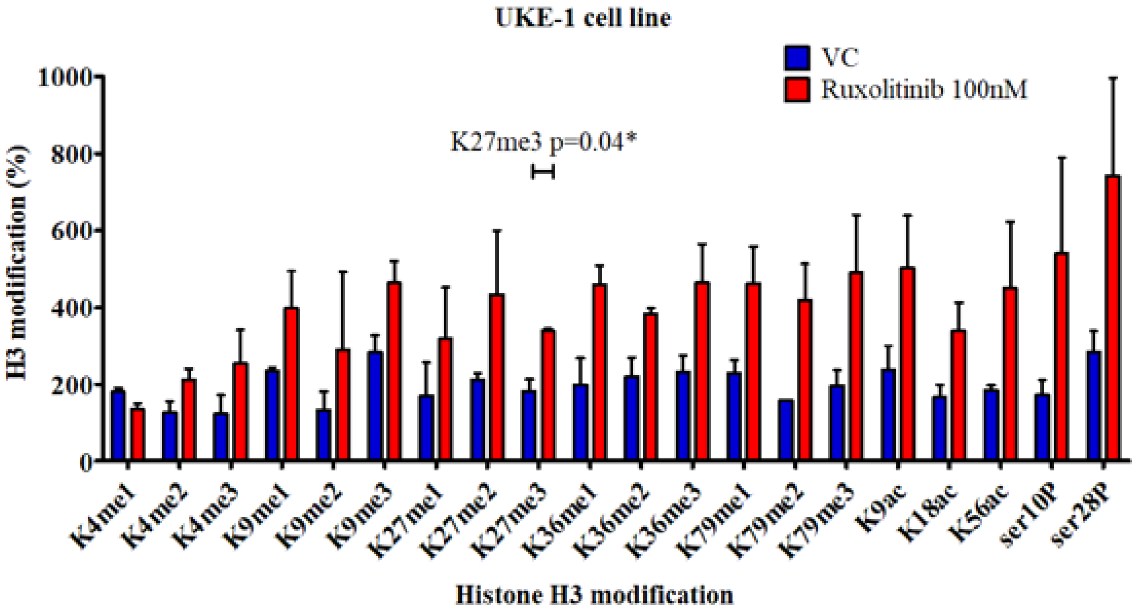
UKE-1 cell line
VC
Ruxolitinib 100nM
K27me3 p=0.04*
H3 modification (%)
0
200
400
600
800
1000
K4me1
K4me2
K4me3
K9me1
K9me2
K9me3
K27me1
K27me2
K27me3
K36me1
K36me2
K36me3
K79me1
K79me2
K79me3
K9ac
K18ac
K56ac
ser10P
ser28P
Histone H3 modification

¶

¶

¶

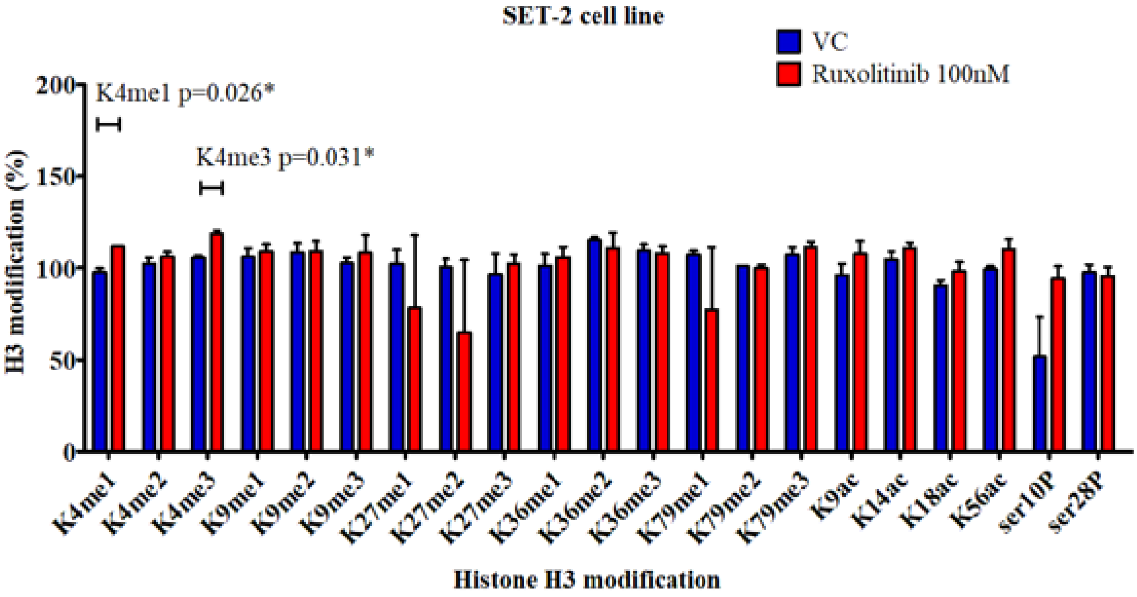
SET-2 cell line
VC
Ruxolitinib 100nM
K4me1 p=0.026*
K4me3 p=0.031*
H3 modification (%)
0
50
100
150
200
K4me1
K4me2
K4me3
K9me1
K9me2
K9me3
K27me1
K27me2
K27me3
K36me1
K36me2
K36me3
K79me1
K79me2
K79me3
K9ac
K14ac
K18ac
K56ac
ser10P
ser28P
Histone H3 modification

¶

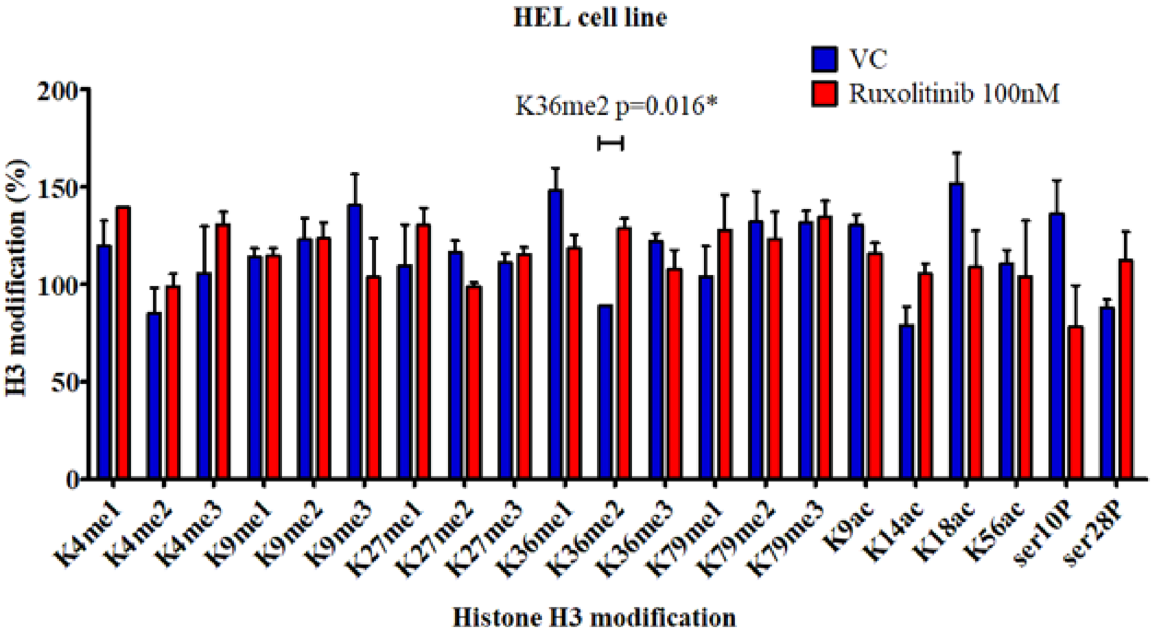

Figure 3. Quantity of H3 modification as a percentage of total H3 present in UKE-1, SET-2 and HEL cell lines. Experiments repeated in duplicate for each cell line and treatment/control.

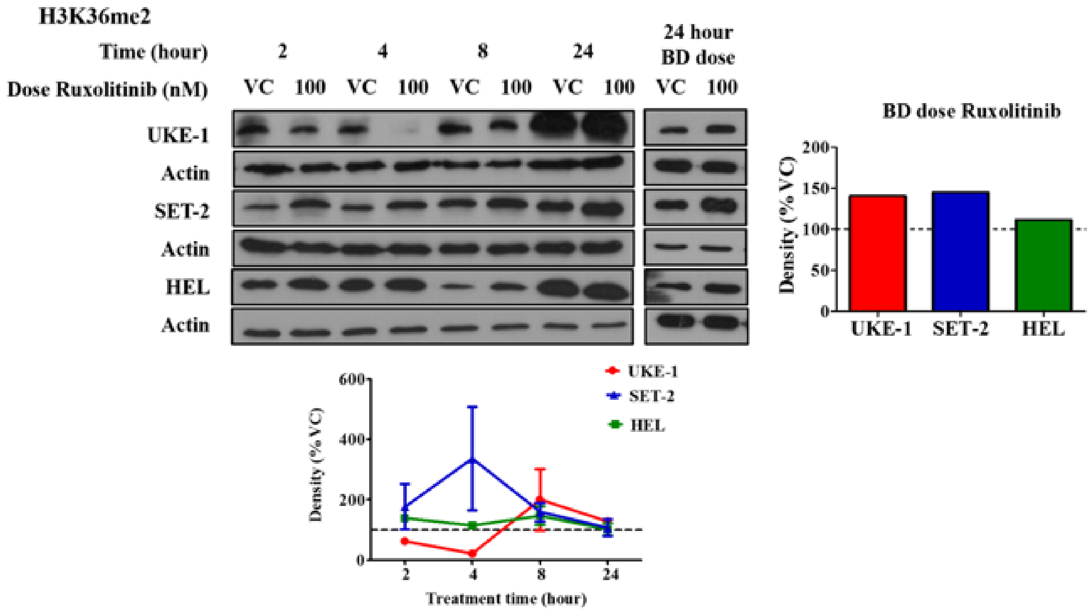
H3K36me2
Time (hour)
2
4
8
24
24 hour
BD dose
Dose Ruxolitinib (nM)
VC 100 VC 100 VC 100 VC 100 VC 100
UKE-1
Actin
SET-2
Actin
HEL
Actin
BD dose Ruxolitinib
Density (% VC)
UKE-1
SET-2
HEL
Density (% VC)
Treatment time (hour)

¶

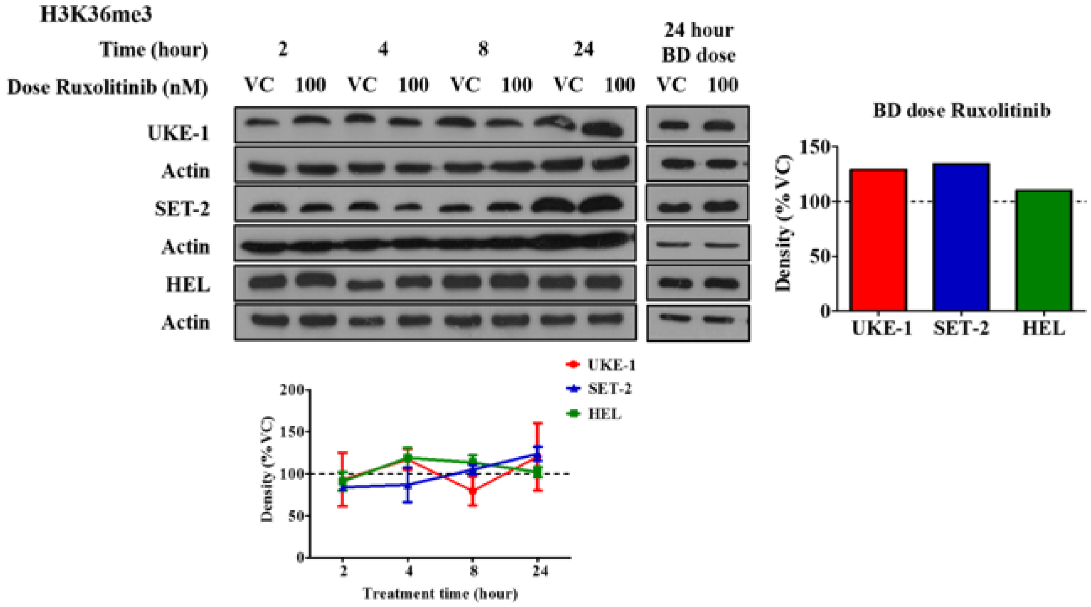
H3K36me3
Time (hour)
2
4
8
24
24 hour
BD dose
Dose Ruxolitinib (nM)
VC 100 VC 100 VC 100 VC 100 VC 100
UKE-1
Actin
SET-2
Actin
HEL
Actin
BD dose Ruxolitinib
Density (% VC)
UKE-1
SET-2
HEL
Density (% VC)
Treatment time (hour)

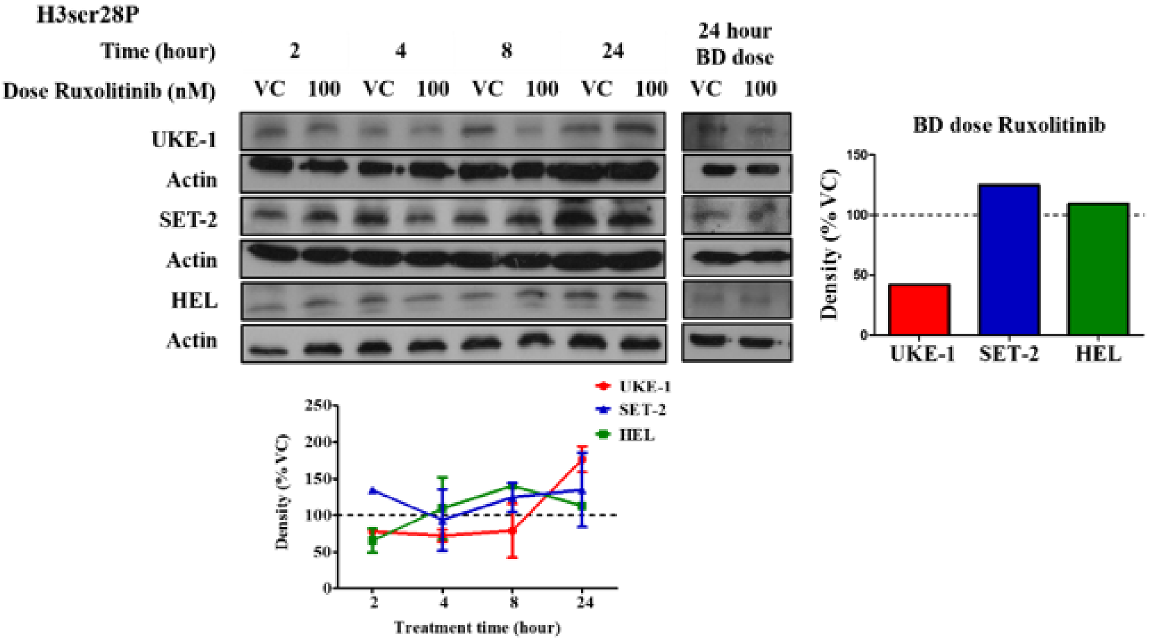
H3ser28P
Time (hour)
2
4
8
24
24 hour BD dose
Dose Ruxolitinib (nM)
VC 100 VC 100 VC 100 VC 100 VC 100
UKE-1
Actin
SET-2
Actin
HEL
Actin
BD dose Ruxolitinib
Density (% VC)
UKE-1
SET-2
HEL
Treatment time (hour)

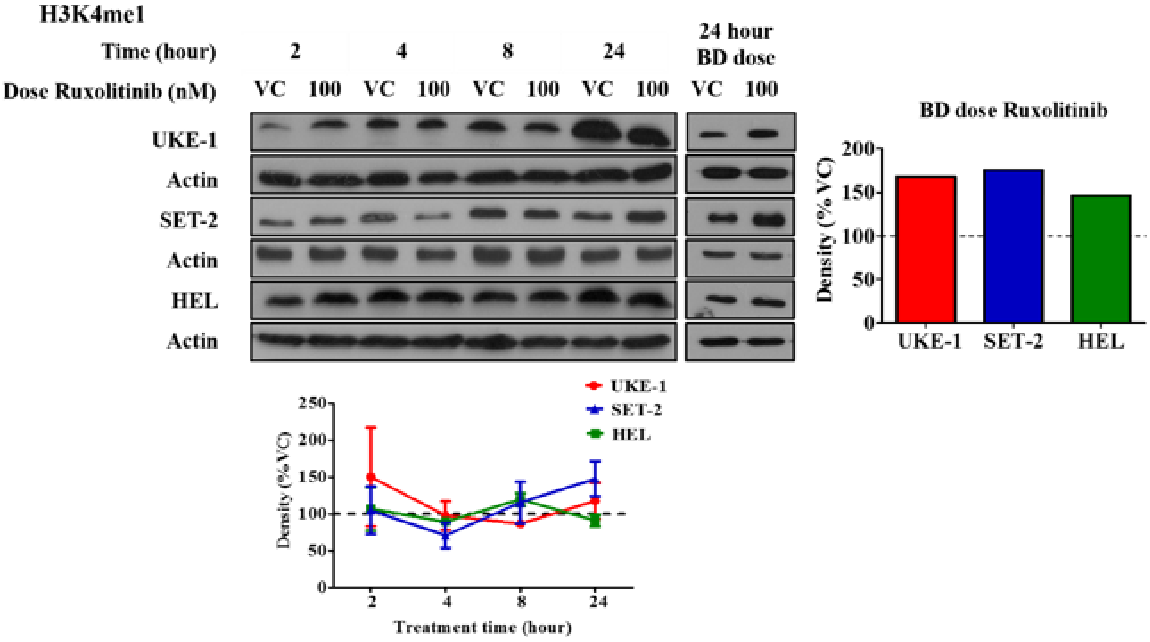
H3K4me1
Time (hour)
2
4
8
24
24 hour BD dose
Dose Ruxolitinib (nM)
VC 100 VC 100 VC 100 VC 100 VC 100
UKE-1
Actin
SET-2
Actin
HEL
Actin
BD dose Ruxolitinib
Density (% VC)
UKE-1
SET-2
HEL
Treatment time (hour)

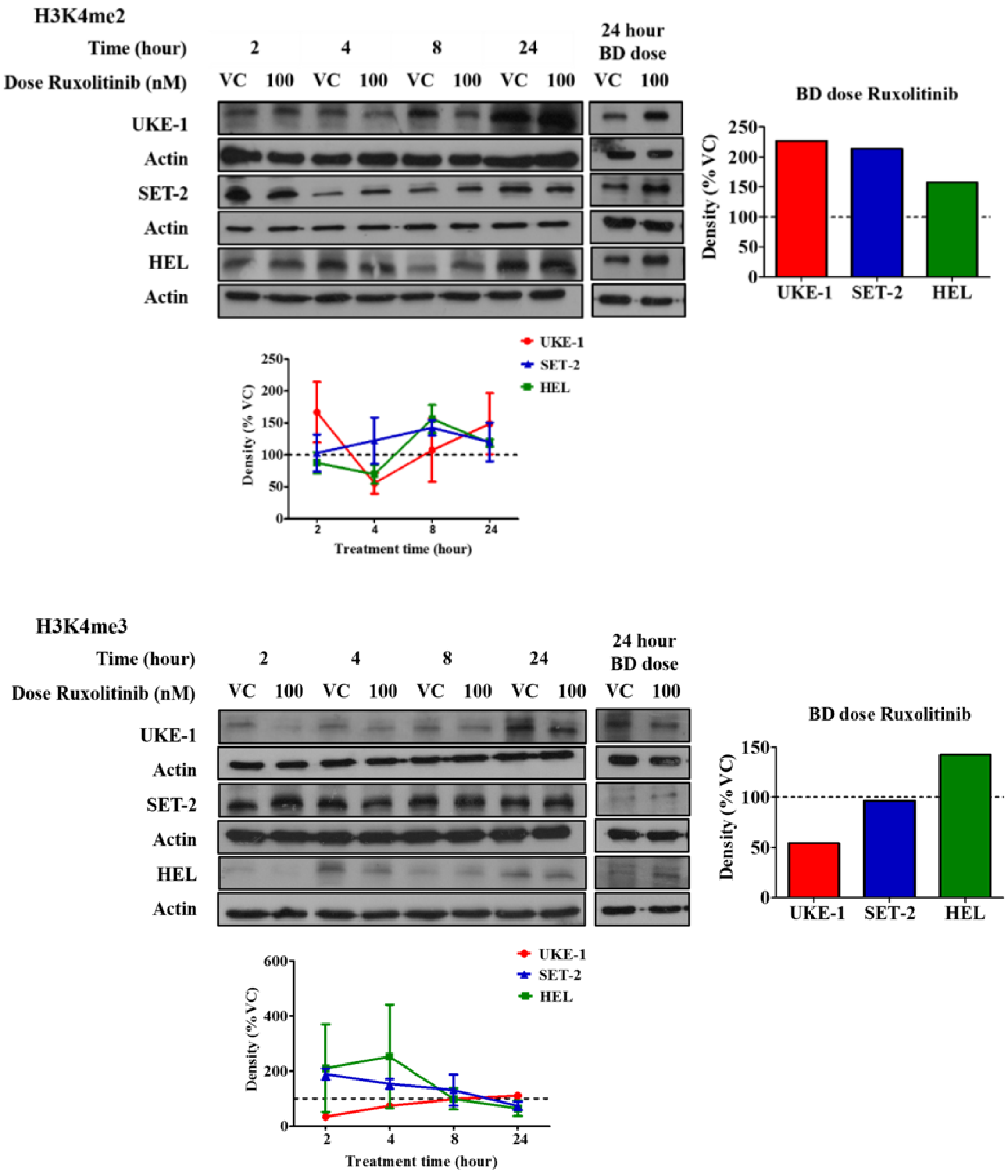

Figure 4. Western blots for H3K36me2, H3K36me3, H3ser28P, H3K4me1, H3K4me2 and H3K4me3 Histone H3 modifications in MPN cell lines (UKE-1, SET-2 and HEL) treated with vehicle control or 100nM Ruxolitinib for 2, 4, 8 or 24 hours (once or twice daily). Densitometry values are normalized for loading control.

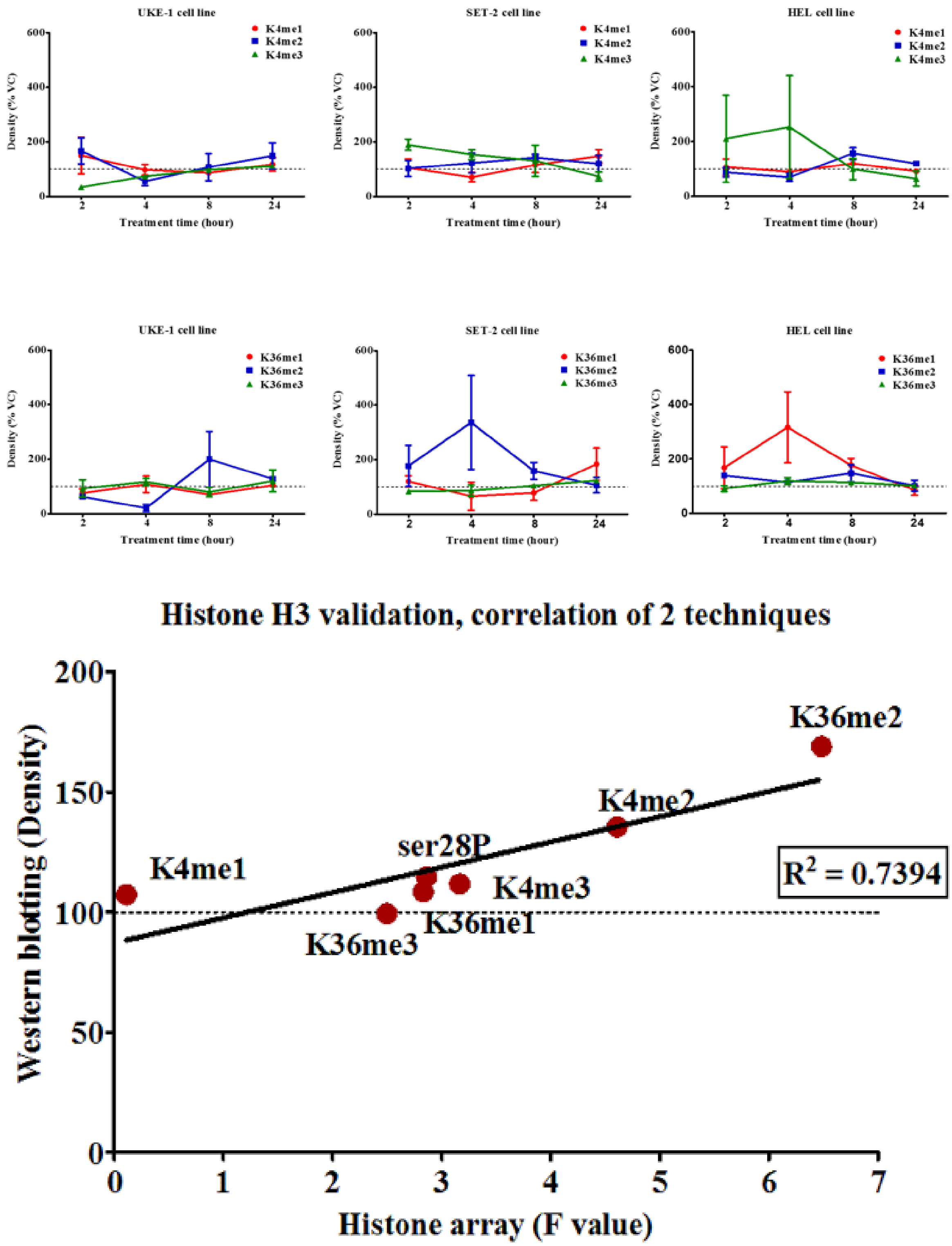

Figure 5. **(A)** Pattern of Histone H3 modifications in MPN cell lines with varying duration of Ruxolitinib treatment. **(B)** Densitometry values from western blots correlated with histone array values for each histone H3 modification tested. A positive correlation is seen between the 2 techniques with an R^2 value of 0.74.

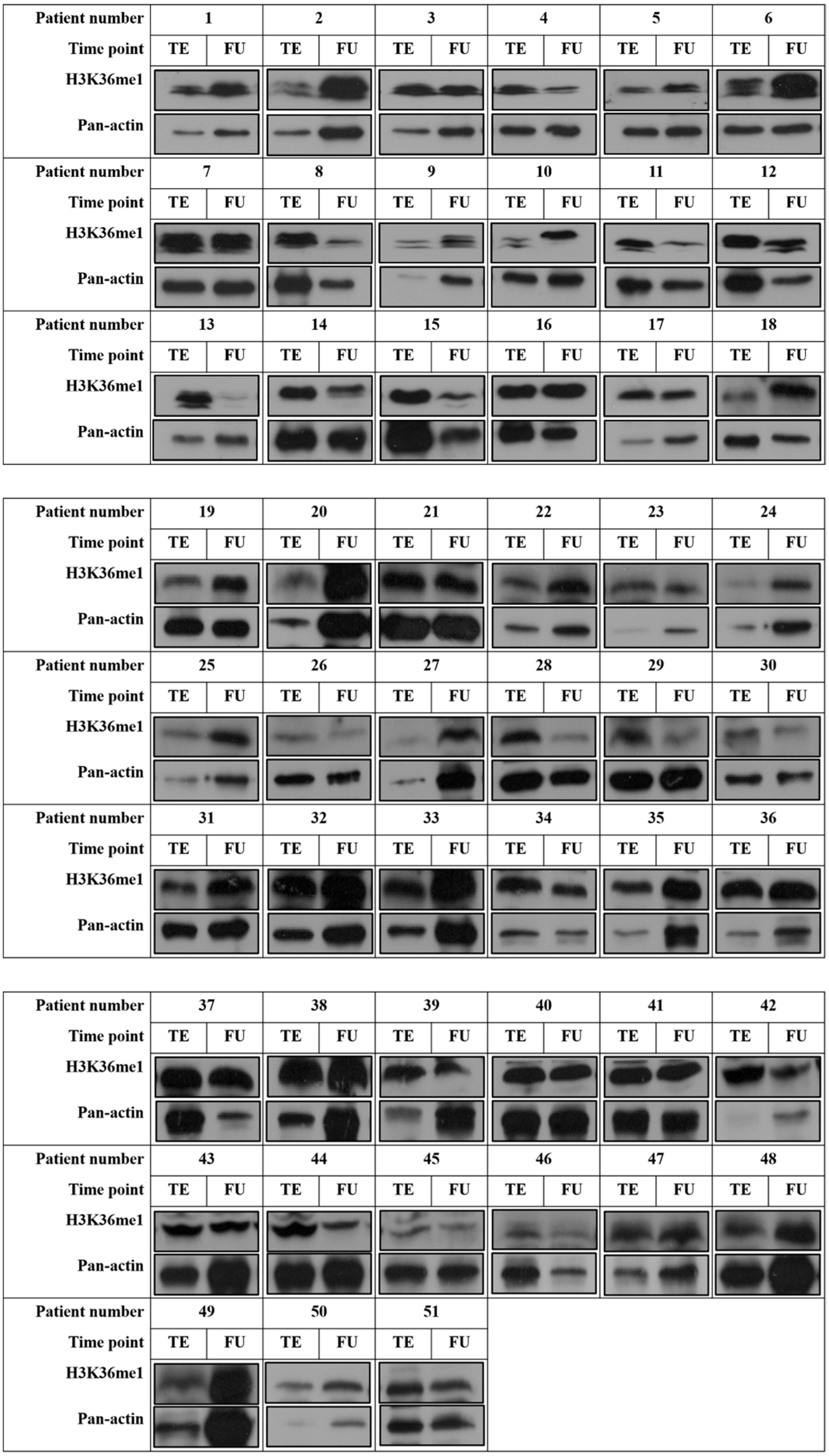
Patient number
1 2 3 4 5 6
Time point
TE FU TE FU TE FU TE FU TE FU TE FU
H3K36me1
Pan-actin
Patient number
7 8 9 10 11 12
Time point
TE FU TE FU TE FU TE FU TE FU TE FU
H3K36me1
Pan-actin
Patient number
13 14 15 16 17 18
Time point
TE FU TE FU TE FU TE FU TE FU TE FU
H3K36me1
Pan-actin
Patient number
19 20 21 22 23 24
Time point
TE FU TE FU TE FU TE FU TE FU TE FU
H3K36me1
Pan-actin
Patient number
25 26 27 28 29 30
Time point
TE FU TE FU TE FU TE FU TE FU TE FU
H3K36me1
Pan-actin
Patient number
31 32 33 34 35 36
Time point
TE FU TE FU TE FU TE FU TE FU TE FU
H3K36me1
Pan-actin
Patient number
37 38 39 40 41 42
Time point
TE FU TE FU TE FU TE FU TE FU TE FU
H3K36me1
Pan-actin
Patient number
43 44 45 46 47 48
Time point
TE FU TE FU TE FU TE FU TE FU TE FU
H3K36me1
Pan-actin
Patient number
49 50 51
Time point
TE FU TE FU TE FU
H3K36me1
Pan-actin

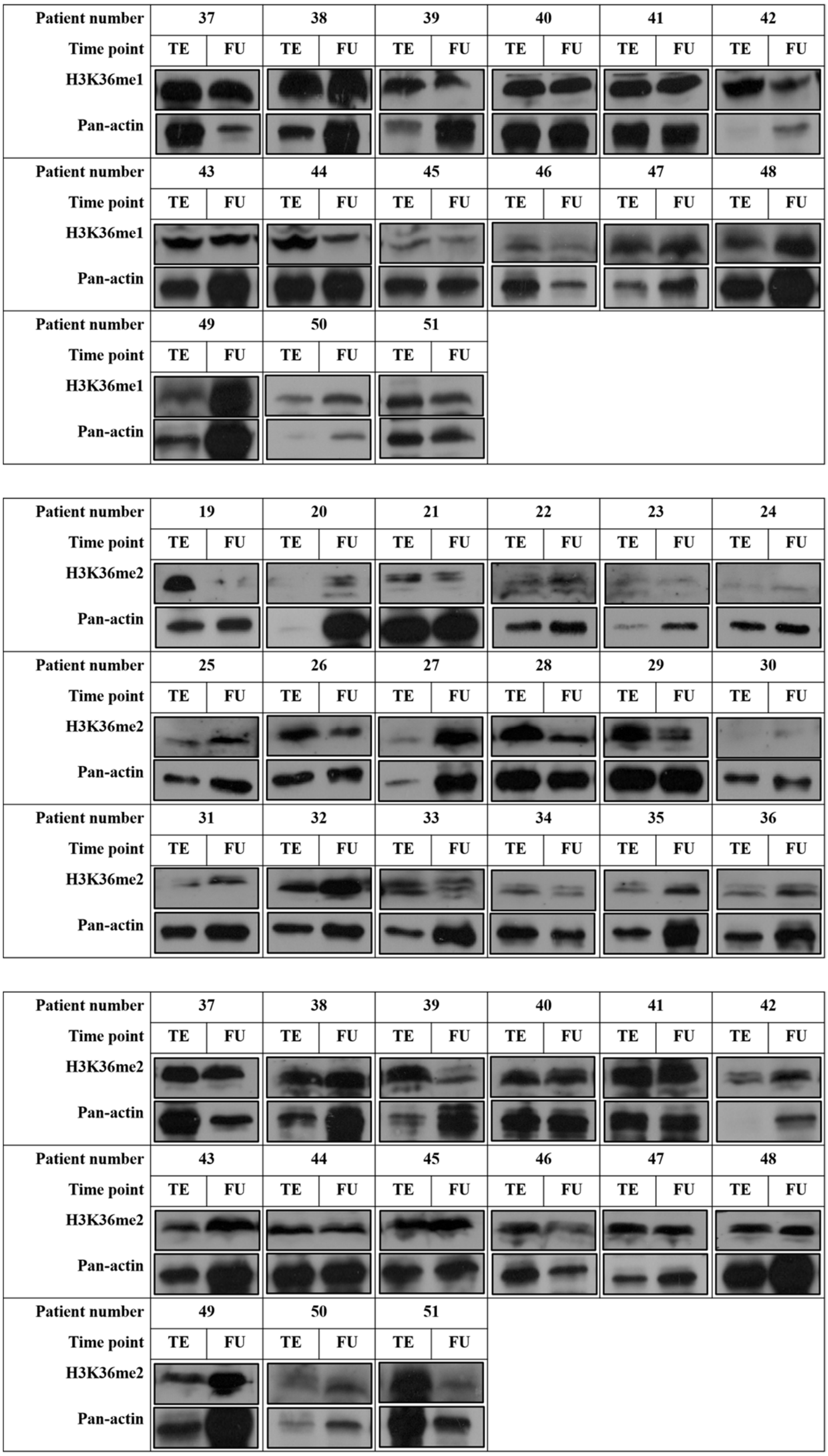
Patient number 37 38 39 40 41 42
Time point TE FU TE FU TE FU TE FU TE FU TE FU
H3K36me1
Pan-actin
Patient number 43 44 45 46 47 48
Time point TE FU TE FU TE FU TE FU TE FU TE FU
H3K36me1
Pan-actin
Patient number 49 50 51
Time point TE FU TE FU TE FU
H3K36me1
Pan-actin
Patient number 19 20 21 22 23 24
Time point TE FU TE FU TE FU TE FU TE FU TE FU
H3K36me2
Pan-actin
Patient number 25 26 27 28 29 30
Time point TE FU TE FU TE FU TE FU TE FU TE FU
H3K36me2
Pan-actin
Patient number 31 32 33 34 35 36
Time point TE FU TE FU TE FU TE FU TE FU TE FU
H3K36me2
Pan-actin
Patient number 37 38 39 40 41 42
Time point TE FU TE FU TE FU TE FU TE FU TE FU
H3K36me2
Pan-actin
Patient number 43 44 45 46 47 48
Time point TE FU TE FU TE FU TE FU TE FU TE FU
H3K36me2
Pan-actin
Patient number 49 50 51
Time point TE FU TE FU TE FU
H3K36me2
Pan-actin

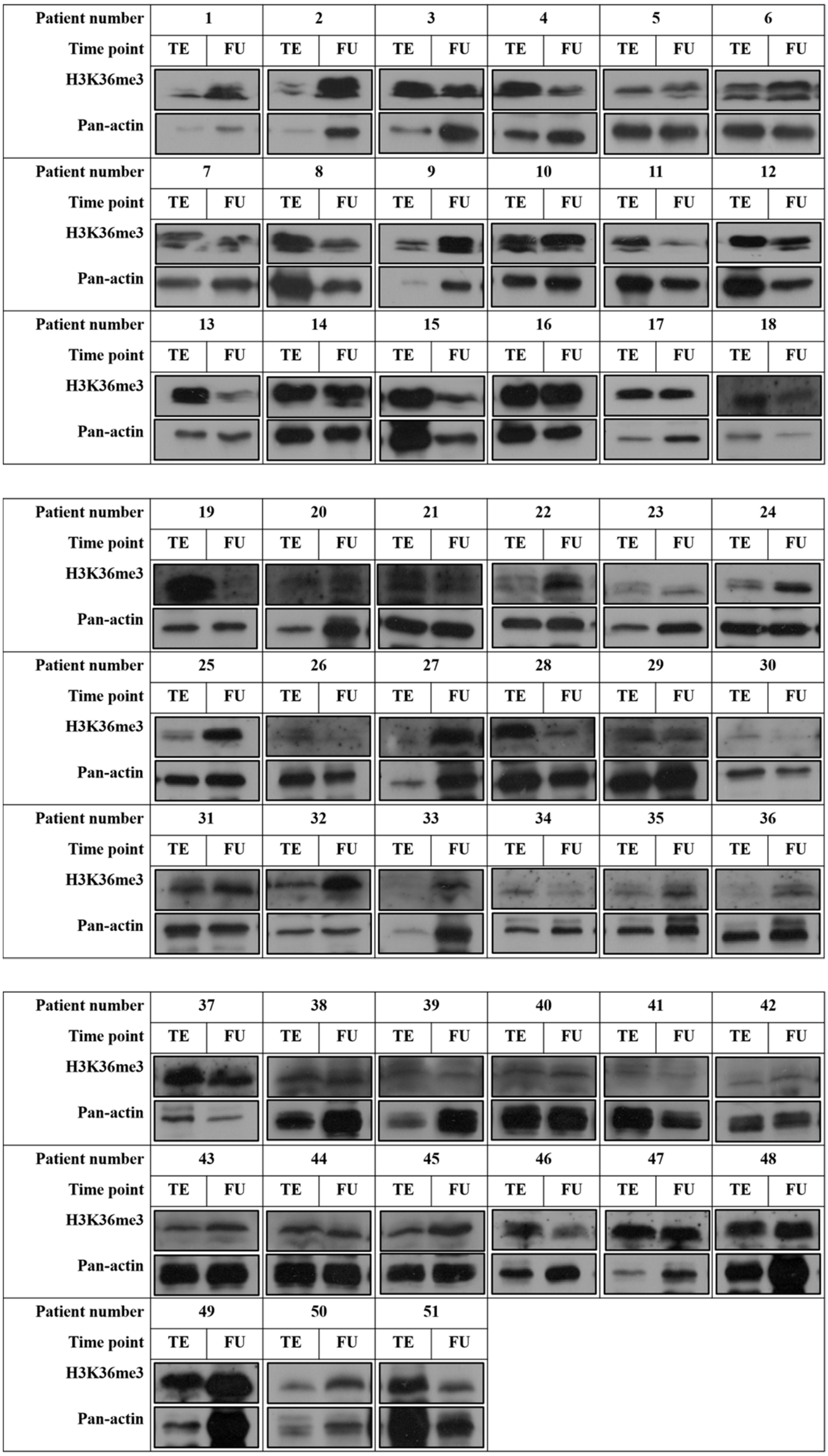

Patient number
1 2 3 4 5 6
7 8 9 10 11 12
13 14 15 16 17 18
19 20 21 22 23 24
25 26 27 28 29 30
31 32 33 34 35 36
37 38 39 40 41 42
43 44 45 46 47 48
49 50 51
Time point
TE FU
H3K36me3
Pan-actin

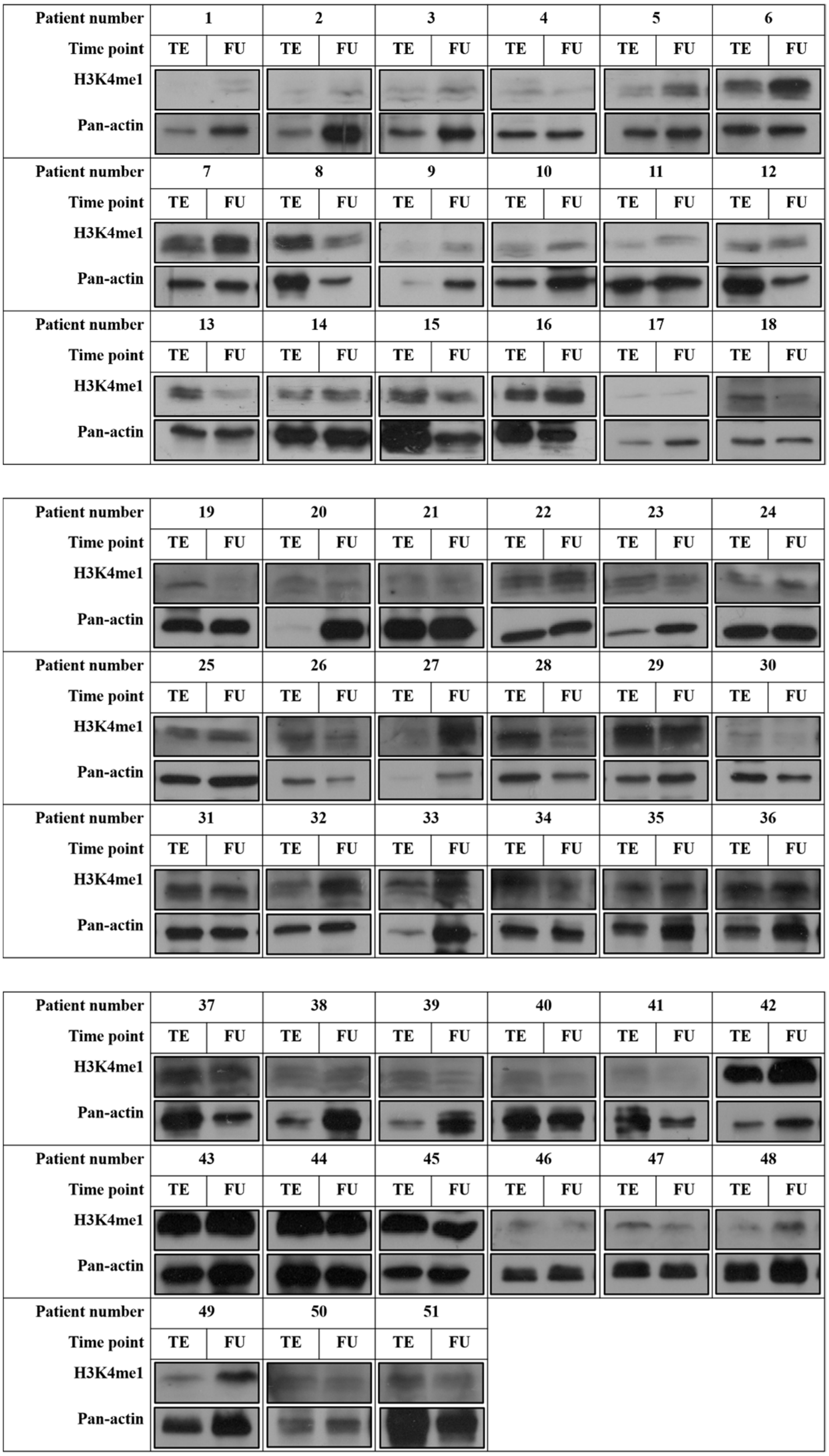
Patient number
1 2 3 4 5 6
7 8 9 10 11 12
13 14 15 16 17 18
19 20 21 22 23 24
25 26 27 28 29 30
31 32 33 34 35 36
37 38 39 40 41 42
43 44 45 46 47 48
49 50 51
Time point
TE FU
H3K4me1
Pan-actin

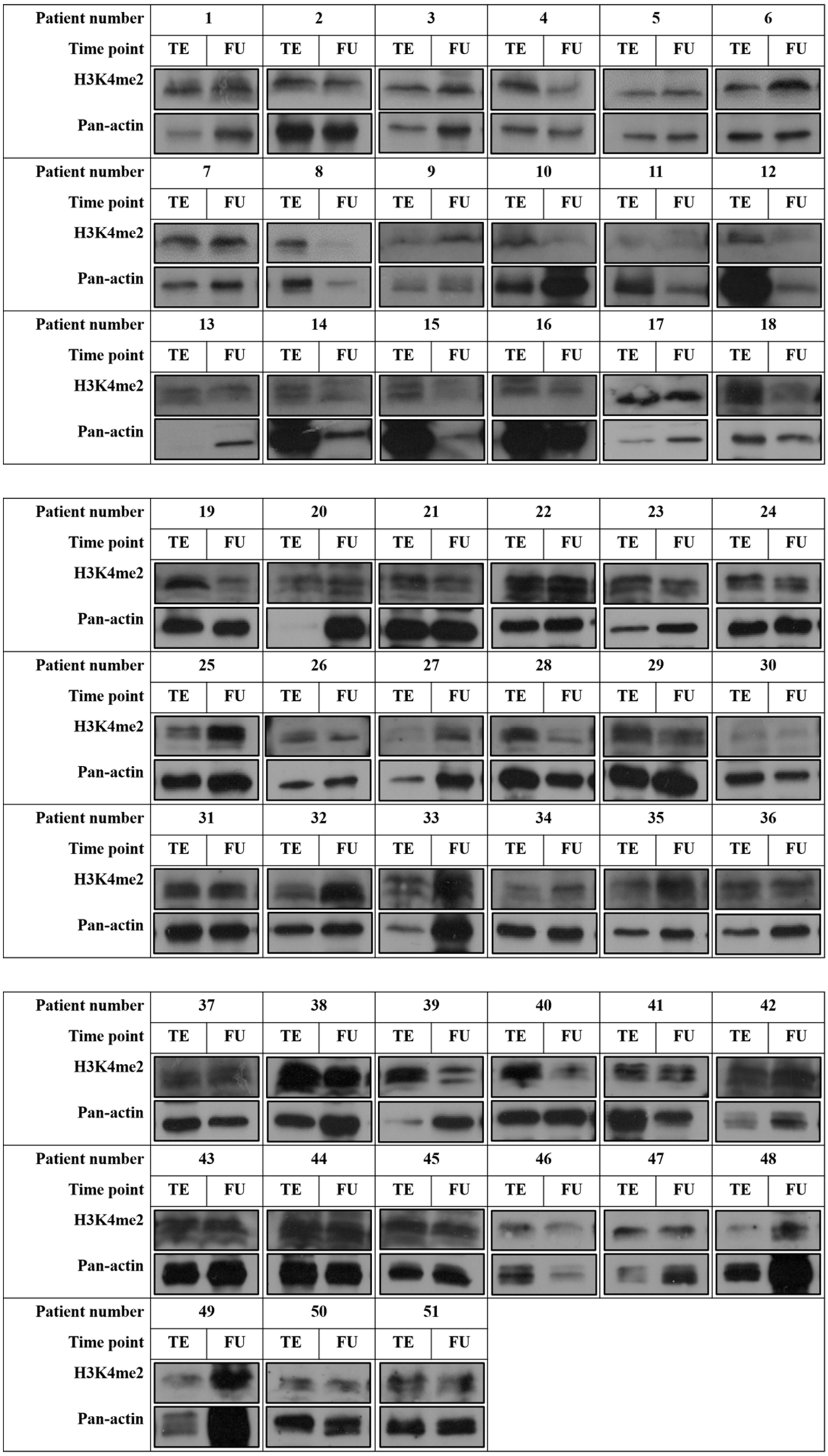
Patient number
1 2 3 4 5 6
Time point
TE FU TE FU TE FU TE FU TE FU TE FU
H3K4me2
Pan-actin
Patient number
7 8 9 10 11 12
Time point
TE FU TE FU TE FU TE FU TE FU TE FU
H3K4me2
Pan-actin
Patient number
13 14 15 16 17 18
Time point
TE FU TE FU TE FU TE FU TE FU TE FU
H3K4me2
Pan-actin
Patient number
19 20 21 22 23 24
Time point
TE FU TE FU TE FU TE FU TE FU TE FU
H3K4me2
Pan-actin
Patient number
25 26 27 28 29 30
Time point
TE FU TE FU TE FU TE FU TE FU TE FU
H3K4me2
Pan-actin
Patient number
31 32 33 34 35 36
Time point
TE FU TE FU TE FU TE FU TE FU TE FU
H3K4me2
Pan-actin
Patient number
37 38 39 40 41 42
Time point
TE FU TE FU TE FU TE FU TE FU TE FU
H3K4me2
Pan-actin
Patient number
43 44 45 46 47 48
Time point
TE FU TE FU TE FU TE FU TE FU TE FU
H3K4me2
Pan-actin
Patient number
49 50 51
Time point
TE FU TE FU TE FU
H3K4me2
Pan-actin

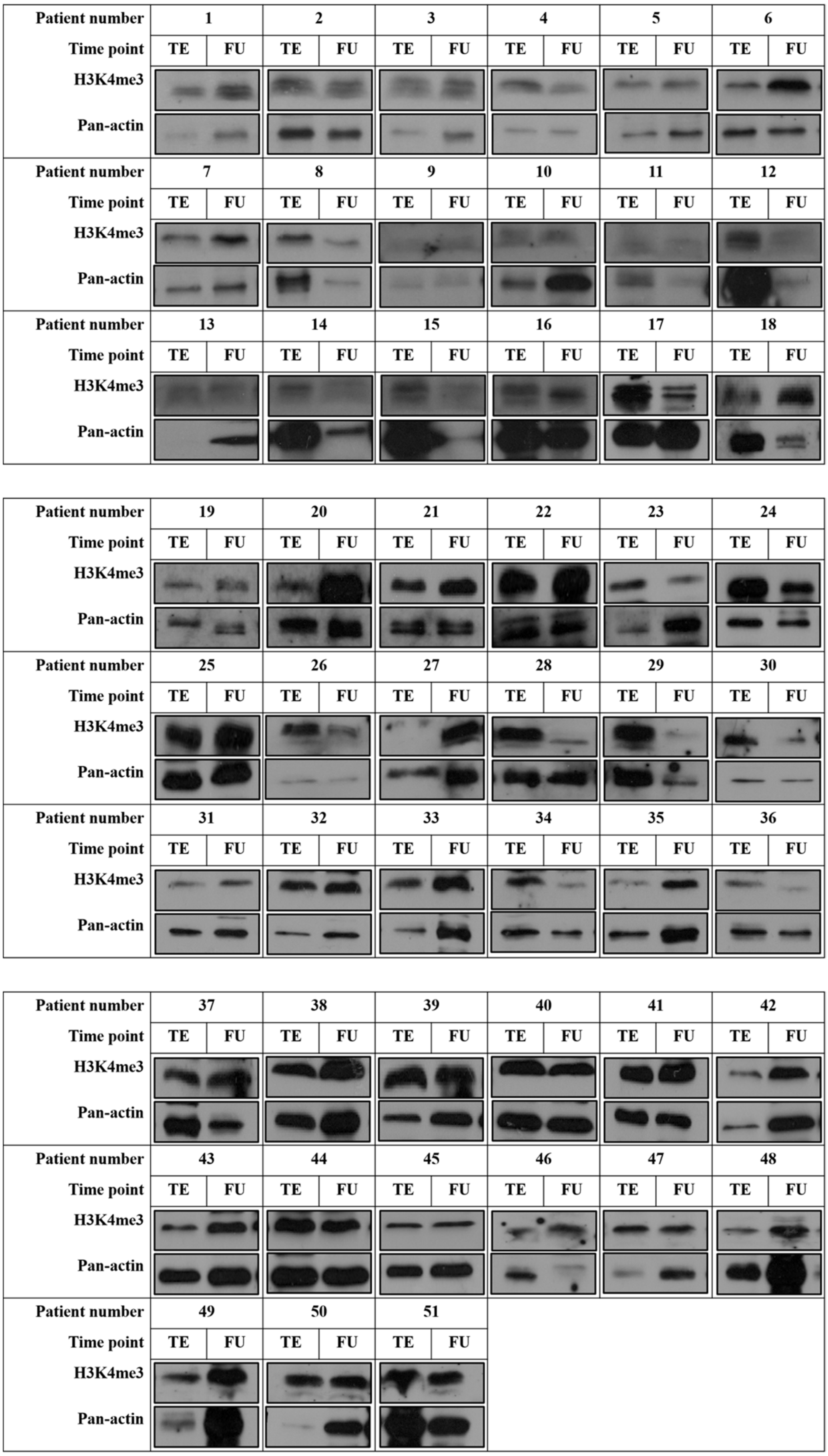

Figure 6. Western blots for mono, di and tri-methylation at lysine 36 and lysine 4 on histone H3 and corresponding pan-actin for all 51 MAJIC patients examined.

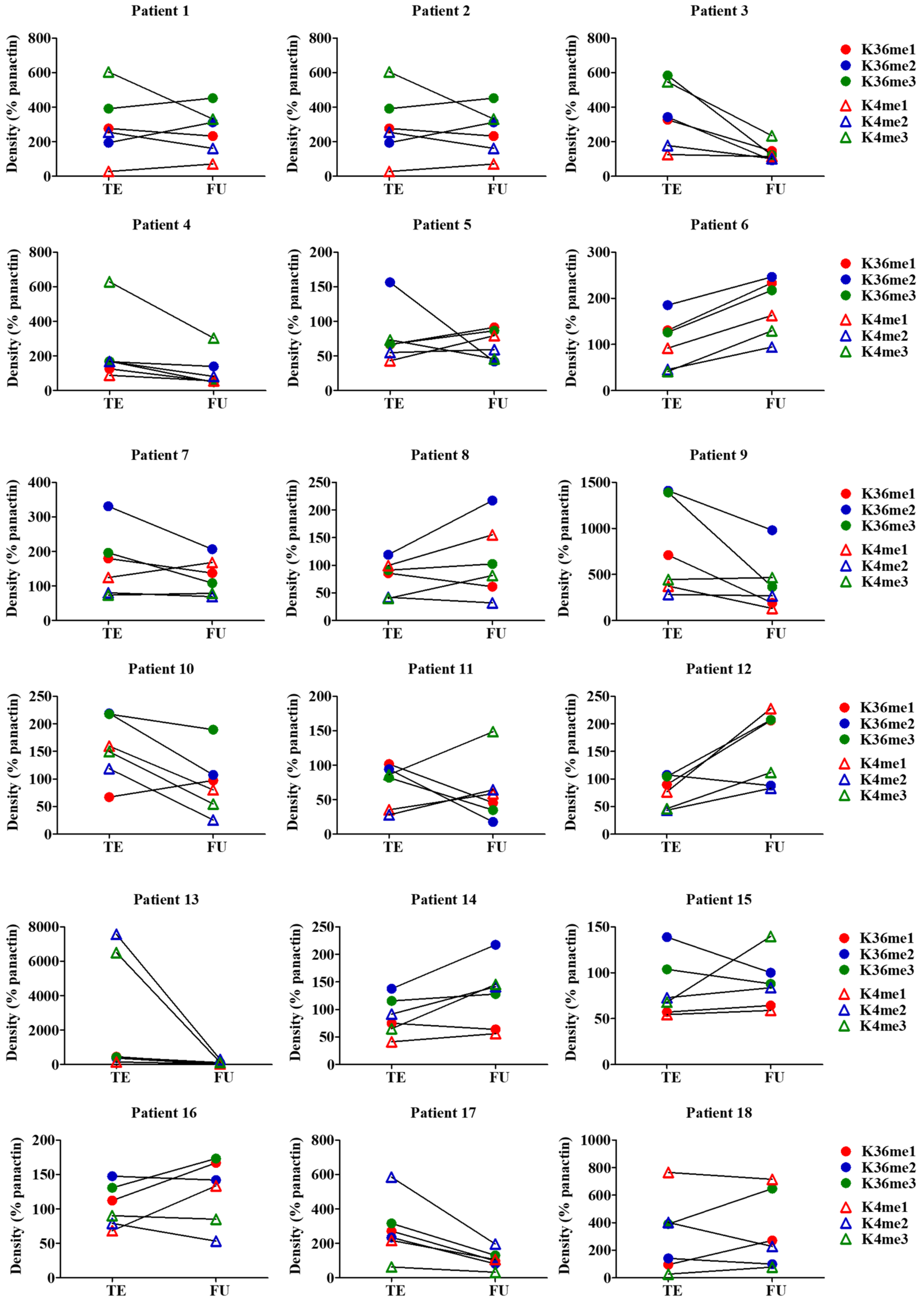
Patient 1
Patient 2
Patient 3
Patient 4
Patient 5
Patient 6
Patient 7
Patient 8
Patient 9
Patient 10
Patient 11
Patient 12
Patient 13
Patient 14
Patient 15
Patient 16
Patient 17
Patient 18
Density (% panactin)
Density (% panacatin)
TE
FU
K36me1
K36me2
K36me3
K4me1
K4me2
K4me3

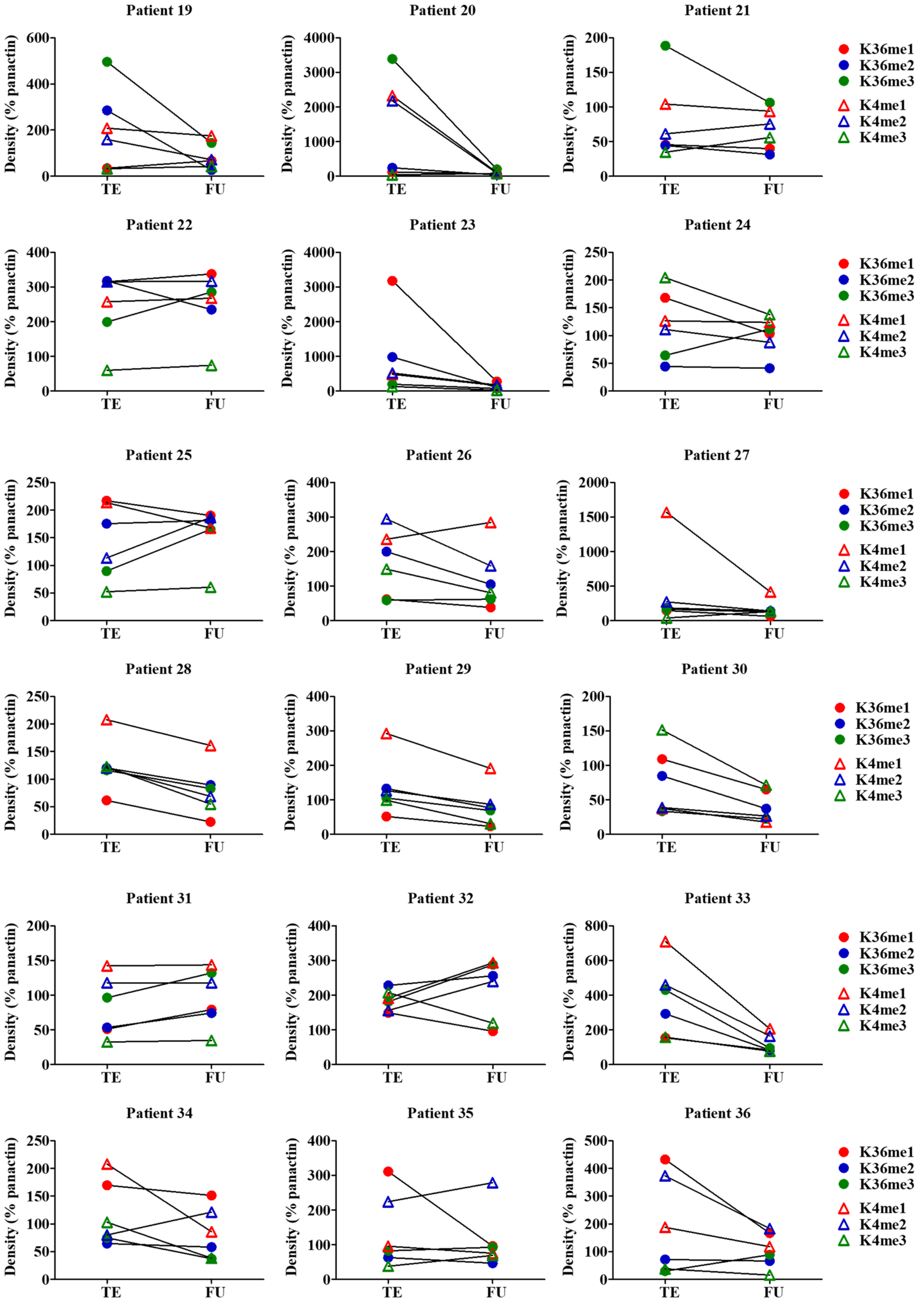
Patient 19
Patient 20
Patient 21
Patient 22
Patient 23
Patient 24
Patient 25
Patient 26
Patient 27
Patient 28
Patient 29
Patient 30
Patient 31
Patient 32
Patient 33
Patient 34
Patient 35
Patient 36
Density (% panactin)
TE
FU
K36me1
K36me2
K36me3
K4me1
K4me2
K4me3

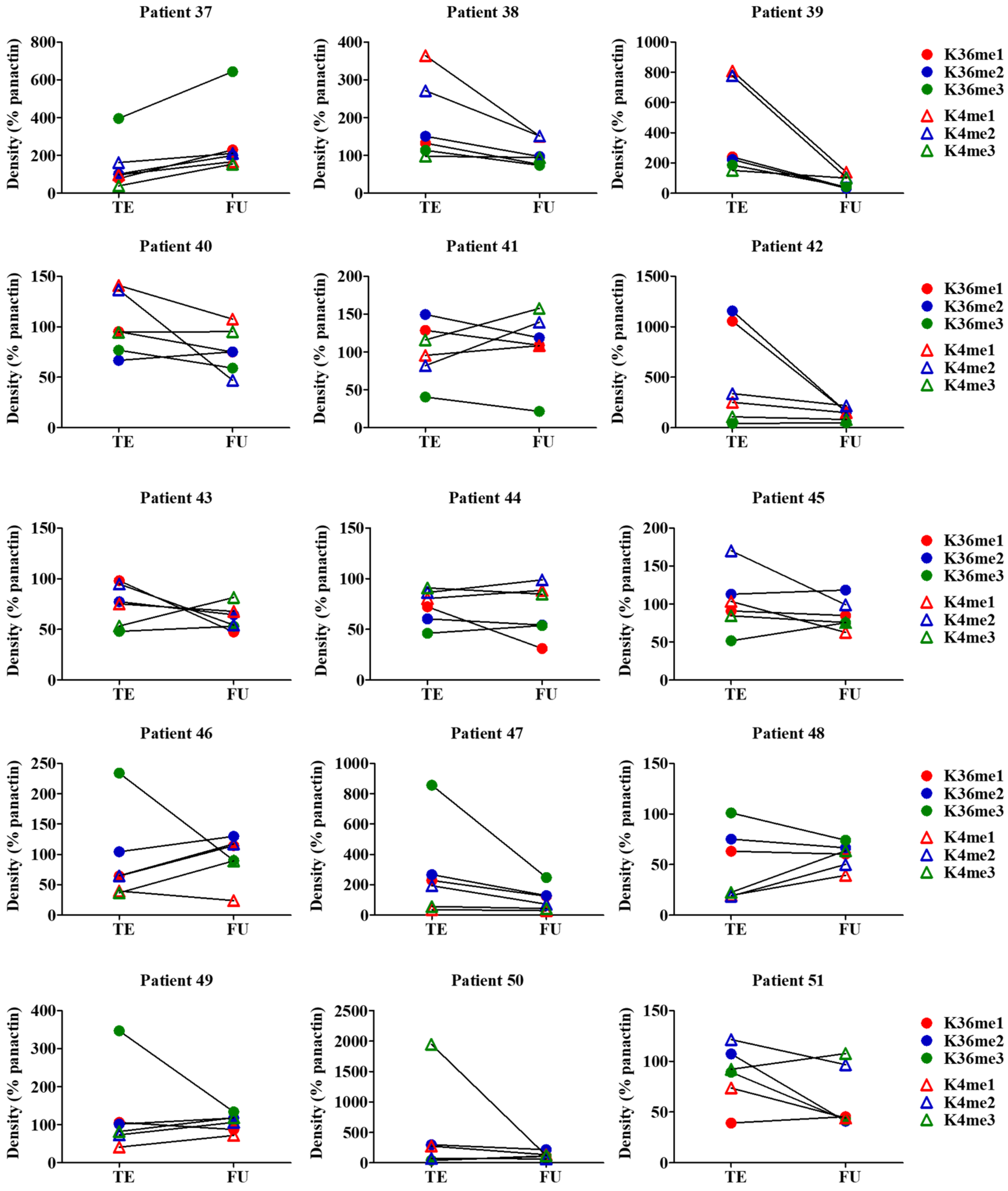

Figure S7: For each patient the normalized densitometry values before and after therapy for each histone mark examine

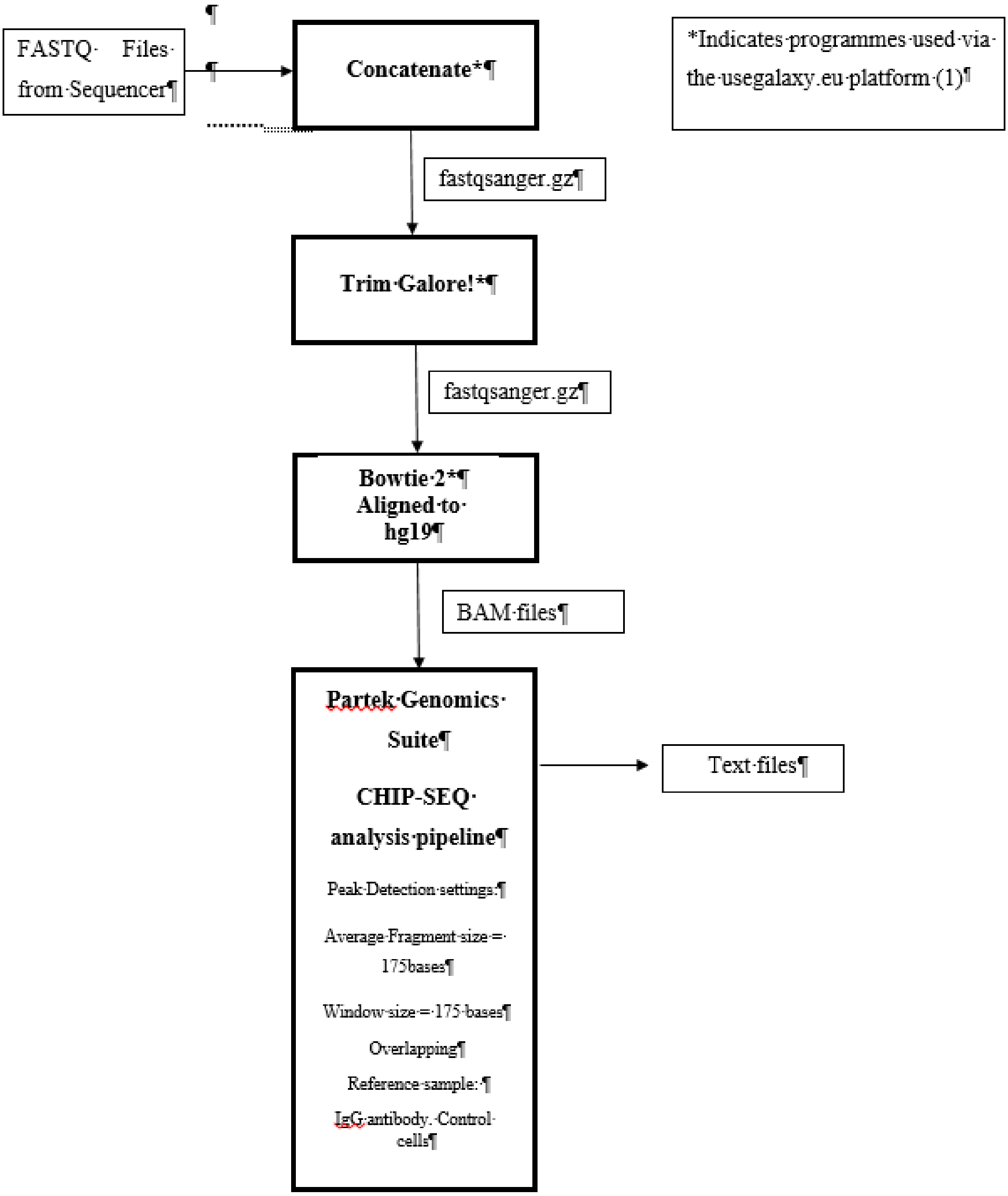

Figure 8. CHIP-seq analysis pipeline.

cancers

MDPI

Table 1. Histone arrays, rank order of *p* values.

Histone modification	UKE-1		SET-2		HEL		All cell lines		UKE-1 + SET-2		UKE-1 + HEL		SET-2 + HEL		Sum of Ranks	Overall rank
	p value	Rank	*p* value	Rank	*p* value	Rank	*p* value	Rank	*p* value	Rank	*p* value	Rank	*p* value	Rank		
**H3K4me1**	0.148	5	0.026	1	0.263	9	0.745	21	0.328	17	0.468	20	0.021	1	74	**12**
**H3K4me2**	0.166	7	0.498	11	0.443	14	0.057	3	0.106	5	0.072	3	0.231	7	50	**3**
**H3K4me3**	0.326	17	0.032	2	0.418	12	0.106	4	0.183	14	0.154	8	0.117	3	60	**5**
**H3K9me1**	0.230	13	0.671	16	0.954	20	0.139	9	0.154	11	0.162	10	0.644	15	94	**17**
**H3K9me2**	0.531	20	0.975	21	0.966	21	0.393	19	0.421	19	0.421	19	0.944	21	140	**21**
**H3K9me3**	0.136	4	0.629	13	0.284	10	0.198	13	0.095	4	0.238	13	0.314	11	68	**6**
**H3K27me1**	0.447	19	0.613	12	0.461	15	0.342	17	0.441	20	0.275	16	0.939	20	119	**20**
**H3K27me2**	0.317	16	0.466	8	0.119	2	0.355	18	0.331	18	0.271	15	0.168	5	82	**15**
**H3K27me3**	0.040	1	0.687	18	0.560	16	0.046	2	0.053	1	0.056	2	0.417	12	52	**4**
**H3K36me1**	0.092	3	0.637	14	0.160	4	0.123	7	0.079	2	0.145	7	0.243	8	45	**2**
**H3K36me2**	0.084	2	0.671	17	0.016	1	0.029	1	0.095	3	0.020	1	0.124	4	29	**1**
**H3K36me3**	0.167	8	0.825	20	0.320	11	0.145	10	0.127	7	0.157	9	0.212	6	71	**10**
**H3K79me1**	0.150	6	0.472	9	0.423	13	0.130	8	0.194	15	0.078	4	0.883	19	74	**13**
**H3K79me2**	0.354	18	0.667	15	0.716	17	0.231	15	0.224	16	0.254	14	0.600	14	109	**19**
**H3K79me3**	0.204	10	0.491	10	0.806	18	0.123	6	0.139	9	0.142	6	0.477	13	72	**11**
**H3K9ac**	0.218	11	0.323	6	0.193	6	0.147	11	0.134	8	0.185	11	0.855	17	70	**7**
**H3K14ac**		21	0.352	7	0.135	3	0.577	20	0.920	21	0.676	21	0.044	2	95	**18**
**H3K18ac**	0.171	9	0.298	5	0.218	7	0.240	16	0.106	6	0.295	17	0.276	10	70	**8**
**H3K56ac**	0.266	14	0.202	4	0.845	19	0.166	12	0.168	13	0.204	12	0.876	18	92	**16**
**H3ser10P**	0.283	15	0.200	3	0.170	5	0.208	14	0.146	10	0.296	18	0.774	16	81	**14**
**H3ser28P**	0.220	12	0.783	19	0.252	8	0.122	5	0.159	12	0.132	5	0.246	9	70	**9**

Table 2. STRING pathway analysis of downregulated genes following ruxolitinib administration to HEL cells.(2).

Term Description	observed gene count	background gene count	false discovery rate
PI3K-Akt signaling pathway	13	348	4.00E-05
Transcriptional misregulation in cancer	9	169	9.92E-05
Jak-STAT signaling pathway	8	160	0.00041
Cytokine-cytokine receptor interaction	9	263	0.0013
Focal adhesion	8	197	0.0013
Inflammatory bowel disease (IBD)	5	62	0.0014
Pathways in cancer	12	515	0.0017
HTLV-I infection	8	250	0.0033
Hippo signaling pathway	6	152	0.0068

Th17 cell differentiation	5	102	0.0068
AGE-RAGE signaling pathway in diabetic complications	5	98	0.0068
TNF signaling pathway	5	108	0.008
Human papillomavirus infection	8	317	0.0094
Proteoglycans in cancer	6	195	0.0154
Measles	5	133	0.0158
Wnt signaling pathway	5	143	0.0174
ECM-receptor interaction	4	81	0.0174
Cell adhesion molecules (CAMs)	5	139	0.0174
Primary immunodeficiency	3	37	0.0174
MAPK signaling pathway	7	293	0.0178
MicroRNAs in cancer	5	149	0.0178
Breast cancer	5	147	0.0178
Hematopoietic cell lineage	4	94	0.0204
Malaria	3	47	0.0229
Insulin resistance	4	107	0.0293
Viral myocarditis	3	56	0.0338
Platelet activation	4	123	0.0435
